# Involvement of Matrix Metalloproteinases in COVID-19: Molecular Targets, Mechanisms, and Insights for Therapeutic Interventions

**DOI:** 10.3390/biology12060843

**Published:** 2023-06-10

**Authors:** Rebecca Salomão, Victoria Assis, Ivo Vieira de Sousa Neto, Bernardo Petriz, Nicolas Babault, João Luiz Quaglioti Durigan, Rita de Cássia Marqueti

**Affiliations:** 1 Laboratory of Molecular Analysis, Postgraduate Program in Health and Sciences and Technology, Faculty of Ceilândia, University of Brasilia, Brasilia 72220-275, DF, Brazil; 2 Laboratory of Molecular Analysis, Postgraduate Program in Rehabilitation Sciences, Faculty of Ceilândia, University of Brasilia, Brasilia 72220-275, DF, Brazil; vicassis.assis@gmail.com (V.A.); joaodurigan@gmail.com (J.L.Q.D.); 3 School of Physical Education and Sport of Ribeirão Preto, University of São Paulo, Ribeirão Preto 14040-907, SP, Brazil; ivoneto04@hotmail.com; 4 Graduate Program in Genomic Sciences and Biotechnology, Catholic University of Brasilia, Brasilia 71966-700, DF, Brazil; bernardopetriz@gmail.com; 5 Laboratory of Exercise Molecular Physiology, University Center UDF, Brasília 71966-900, DF, Brazil; 6 INSERM UMR1093-CAPS, UFR des Sciences du Sport, Université de Bourgogne, F-21000 Dijon, France; nicolas.babault@u-bourgogne.fr; 7 Centre d’Expertise de la Performance, UFR des Sciences du Sport, Université de Bourgogne, F-21000 Dijon, France

**Keywords:** extracellular compartment, biomarker, inflammation, diseases, SARS-CoV-2, metallopeptidases

## Abstract

**Simple Summary:**

The costs worldwide of the coronavirus disease 2019 (COVID-19) have been tremendous. With millions of deaths in different countries, understanding biomarkers is essential to diminish the disease burden. For this purpose, a reflective understanding of the pathobiology of COVID-19 is required. During viral infection, some proteins require proteolytic activation and are involved in cell repair and maladaptive organ remodeling. This review summarizes the current findings on the role of increasing matrix metalloproteinases (MMPs) during acute respiratory syndrome coronavirus-2 (SARS-CoV-2) infection. COVID-19 patients present distinct complications that can be distinguished by MMP levels. MMPs in excess can generate tissue damage and aggravate the possible complications of COVID-19. MMPs participate in chronic inflammation, and their abnormal regulation is associated with human diseases. Interestingly, individuals with comorbidities or pathological conditions are more susceptible to increasing MMPs and to developing severe COVID-19 illnesses. Furthermore, MMP levels can predict the risk of in-hospital death by COVID-19, suggesting possible prognostic roles. Extensive knowledge of MMPs could provide novel perspectives on the symptoms, pathogenesis, and treatment of COVID-19.

**Abstract:**

MMPs are enzymes involved in SARS-CoV-2 pathogenesis. Notably, the proteolytic activation of MMPs can occur through angiotensin II, immune cells, cytokines, and pro-oxidant agents. However, comprehensive information regarding the impact of MMPs in the different physiological systems with disease progression is not fully understood. In the current study, we review the recent biological advances in understanding the function of MMPs and examine time-course changes in MMPs during COVID-19. In addition, we explore the interplay between pre-existing comorbidities, disease severity, and MMPs. The reviewed studies showed increases in different MMP classes in the cerebrospinal fluid, lung, myocardium, peripheral blood cells, serum, and plasma in patients with COVID-19 compared to non-infected individuals. Individuals with arthritis, obesity, diabetes, hypertension, autoimmune diseases, and cancer had higher MMP levels when infected. Furthermore, this up-regulation may be associated with disease severity and the hospitalization period. Clarifying the molecular pathways and specific mechanisms that mediate MMP activity is important in developing optimized interventions to improve health and clinical outcomes during COVID-19. Furthermore, better knowledge of MMPs will likely provide possible pharmacological and non-pharmacological interventions. This relevant topic might add new concepts and implications for public health in the near future.

## 1. Introduction

Coronavirus disease 2019 (COVID-19) is characterized as a highly contagious infectious disorder caused by severe acute respiratory syndrome coronavirus 2 (SARS-CoV-2), which requires serious medical attention [1]. According to the World Health Organization (WHO), COVID-19 is a global disease of epidemic proportions that affects children, adolescents, and adults in countries with different levels of development, causing a catastrophic effect on the world’s demographics [2]. To date, there have been 525 million confirmed cases, with more than 6.2 million deaths worldwide, emerging as a global health crisis [2]. The most common symptoms are fever, fatigue, respiratory impairment, dyspnea, and, in more severe cases, acute myocardial injury and multiple organ failure [3]. Despite significant advances in clinical research that have improved understanding of SARS-CoV-2 and COVID-19 management, there is still a lack of knowledge regarding the intricate biological mechanisms that underlie the pathogenesis of the disease. Chronic inflammation, autophagy dysregulation, oxidative stress, and metabolic dysfunction are intertwined processes that play roles in disease progression via the interference of several signaling pathways [4,5]. It has been shown that regardless of the molecular mediator, viral recognition culminates in the increase in pro-inflammatory cytokine production, chemical intermediaries, and apoptosis, to provide an optimal antiviral response [6]. Numerous immune cells, including macrophages, lymphocytes, and neutrophils, migrate to the organs to restrain SARS-CoV-2, which impairs cellular function and overall homeostasis [7]. When this attempt to limit infection is exacerbated, specific oxidative and inflammatory outcomes cause secondary cellular damage to functional and uninfected tissues of the different physiological systems [8].

It has recently been suggested that an inflammatory cascade is possibly induced by matrix metalloproteinases (MMPs) production [8]. The pre-infection level of MMPs or the potential of the host cells to produce these enzymes worsen the COVID-19 disease time-course [8]. MMPs are supposed to contribute to adverse organ remodeling, damage, destructive processes, and tissue pathologies. Recent clinical data indicate an increase in MMPs in the blood circulation of mild, moderate, and severe COVID-19 patients [9,10]. Elsewhere, serum levels of MMPs were significantly higher in COVID-19 patients than in healthy subjects and gradually increased with the disease stage [10,11].

Furthermore, other investigations showed increased gene expression of MMPs in cerebrospinal fluid [12], myocardium [13], and lung [14]. MMPs characterize a family of proteolytic enzymes that contain a zinc ion at the active site of catalysis and cleave a wide diversity of substrates, such as extracellular matrix (ECM) elements (collagens, fibronectin, and elastin), secreted and ECM-anchored growth factors, chemokines, and cytokines [8]. Therefore, assessing different MMPs as biomarkers could help support COVID-19 screening, as prognostic and predictive markers related to drug response.

Given that the clinical relevance of MMPs in COVID-19 is dispersed throughout the literature, our review aims to examine the mechanisms by which excessive activation of MMPs could lead to host damage by disrupting target organs during COVID-19. Our study encompasses a comprehensive range of research designs, including case-control, cross-sectional, longitudinal, and experimental studies, which investigate the impact of COVID-19 on MMPs in multiple organs across diverse patient populations. Initially, we provide the historical framework of the field, after which we focus on revisiting the biological advances in understanding the structure and function of MMPs, as well as the time-course and the progressive changes in MMPs during COVID-19. Accurate and timely diagnosis of the risk of death during COVID-19 infection is critical for effective clinical management. To this end, it is essential to understand the activity of MMPs at a given time or according to the severity of patients affected by the disease. This information could provide valuable insights into the underlying mechanisms driving disease progression and enable clinicians to intervene early with appropriate treatment strategies. By leveraging this knowledge, we can improve outcomes for COVID-19 patients and reduce the overall burden of the disease. In addition, to elucidate the interplay between pre-existing comorbidities or chronic diseases and MMPs, we examine how disease severity can influence MMP activity. Finally, we discuss the potential for translating this knowledge into novel therapeutic approaches. These topics can enhance our comprehension of disease pathogenesis, progression, and recovery, while offering fresh perspectives on achievable treatment objectives.

## 2. MMP Structure and Function

The extracellular matrix (ECM) is a network of macromolecules, composed of collagen, enzymes, and proteins, that provides structural and biochemical support. The ECM has a diverse composition of fibers, proteoglycans, glycoproteins, and polysaccharides which regulate cell migration, growth, and differentiation [15]. The ECM structure plays an important role in supporting organs and tissues, as well as participating in other functions, such as regulation of the cell cycle and cell motility, survival, and apoptosis, in addition to the distribution of growth factors and integration of signals in cells [16]. MMPs are a large family of calcium-dependent zinc-containing endopeptidases with multiple roles in tissue remodeling and the capacity to degrade most components of the ECM, including collagens, elastin, gelatin, matrix glycoproteins, and proteoglycan [16,17]. MMPs also participate in different biological and physiological processes that are regulated by hormones, growth factors, and cytokines [16,17].

Since Jerome Gross and Charles Lapiere discovered the first MMP in 1962, MMPs have been shown to be an extremely relevant family of proteinases, extensively studied in many animal and tissue models [18]. MMPs have also been recognized as biomarkers in several fields, such as diagnosis, monitoring, and treatment efficacy. After the identification of additional MMPs, these proteases were identified by different names, according to the classification by substrate of consumption [19]. Ref. [19] According to Cui and Khalil [20], the MMP family is composed of 28 members, of which 23 are expressed in human tissues, and 14 of these are represented in the vascular system [20].

The biological role of MMPs is the cleavage of ECM substrates (proteins, glycoproteins, membrane receptors, cytokines, and growth factors). Based on their structural domain, MMPs can be classified into several categories, including membrane-type matrix metalloproteases (MT-MMPs), collagenases, gelatinases, stromelysins, matrilysins, and other MMPs [16,20].

In addition to the extracellular functions already well described in the literature involving ECM degradation, most MMPs also have intracellular functions [21,22]. The intracellular presence of MMPs has been detected in different cell types in mammals [23,24,25]. They have been found in the cytosol [26,27], within the nucleus [28,29], and within mitochondria [26,27]. However, the mechanisms that generate the intracellular localization of MMPs and their respective functions remain unknown [30].

**Figure 1:**
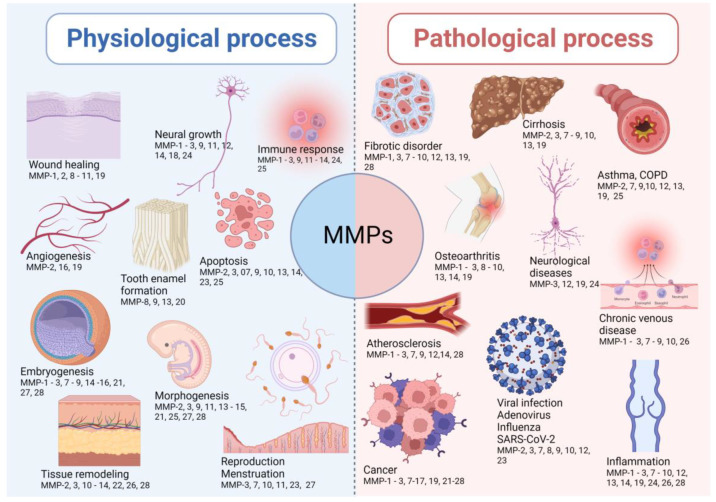
The representative implications of MMPs in both physiological and pathological conditions. The lists of MMPs involved in the presented physiological and pathological processes are not exhaustive, as there are other MMPs involved in the listed processes.

MMPs are implicated in many biological processes, including embryonic development, cell proliferation, migration, and differentiation, angiogenesis, remodeling of ECM, tissue invasion and vascularization, and apoptosis [16,31,32,33,34,35,36,37,38,39,40,41,42,43,44,45,46,47,48,49,50,51,52,53,54,55,56,57,58,59,60,61]. The imbalance between metalloproteinases and their inhibitors leads to the progression of inflammatory and autoimmune diseases. This is highlighted by many spontaneous and induced inflammatory phenotypes in mice lacking one or more metalloproteinases or their inhibitors. The representative implications of MMPs in physiological and pathological conditions are shown in Figure 1.

## 3. Role of COVID-19 in Activating MMPs

The molecular basis of clinical COVID-19 needs to be better explained [10]. It results from a process of interaction between the immune, inflammatory, and coagulative cascades, which are closely intertwined. Angiotensin-converting enzyme 2 (ACE2) has been reported in the literature as a target of SARS-CoV-2 [62,63]. This enzyme has pleiotropic functions that participate in the renin-angiotensin system (RAS) and present a target site for SARS-CoV-2 to mediate its actions [62,63]. In sequence, this virus binds to the host cell receptors through the spike of glycoprotein present in the viral membrane [63,64,65,66]. After binding to the host cell receptor via ligand-receptor interactions, the virus membrane fuses with the host cell membrane, enabling the virus to enter the host cell [67]. As a result, a sudden increase in viral load can trigger an exaggerated immune response, including the expression and proteolytic activation of MMPs, through mechanisms that will be described below [11,68]. Sudden activation of MMPs can disrupt the balance of the extracellular matrix, resulting in tissue damage. Furthermore, increased angiotensin II (ANG II) can promote the expression of MMPs.

**Figure 2:**
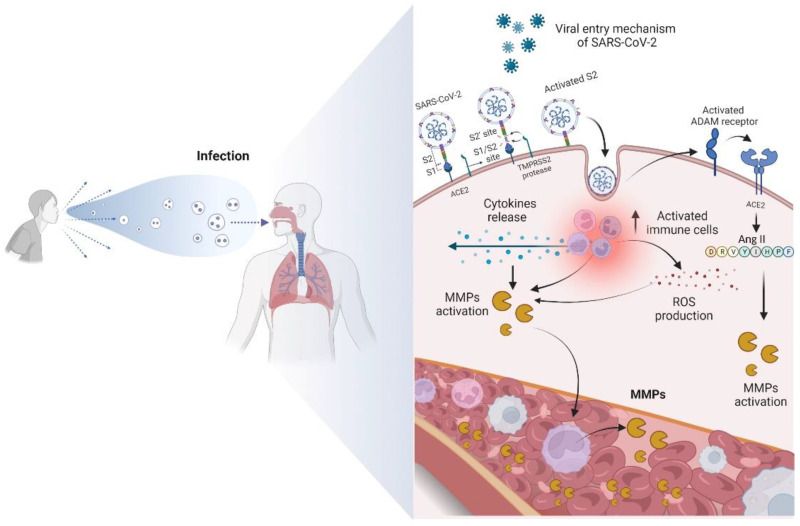
Overview of the main mechanism of entry of SARS-CoV-2 into the cell and the biomolecular changes that occur from contamination. The virus binds to the ACE2 receptor, becoming active. The contact of SARS-CoV-2 with the cell membrane then enables the activation of ADAM receptors, favoring the increase in ANG II and expression of MMPs. The activation of immune cells produces ROS and a cytokine storm, promoting the proteolytic activation of MMPs and consequent increase in the levels of these enzymes in the bloodstream.

ANG II also aids in cytokine storming in patients with COVID-19 by stimulating ADAM-17 enzymatic activity. Consequently, there is an increase in TNF-α levels, increasing NF-kβ activity [69,70]. ADAM-17 is a membrane-bound zinc endopeptidase, containing an active site region similar to that of the MMP family of enzymes. Interestingly, ADAM-17 is associated with the pathogenesis of COVID-19 [8,70]. The relationship between ADAM-17, ACE2 release, and elevated levels of TNF-α was identified in patients with this disease [69,70]. In addition to cytokines, chemokines, and interleukins, MMPs also participate intensely in the cytokine storm, enhancing the destructive effects [8], as shown in Figure 2.

The expression of MMPs in cells in the form of catalytically inactive zymogens is known as pro-MMP. This latent form is maintained through the interaction between a conserved cysteine residue (Cys) in the inhibitory domain and a zinc ion in the catalytic domain [71]. Activation of the MMPs most widely described in the literature occurs during secretion into the cellular space, through the proteolytic removal of the autoinhibitory domain and, consequently, exposure of the catalytic site [38,72,73]. Furthermore, non-proteolytic activation of MMPs occurs by covalent modification of the Cys residue in the autoinhibitory domain by oxidative stress effectors [74].

ROS, such as peroxynitrite and glutathione, have been implicated in this process. For example, the study by Viappiani et al. [71] investigated the activation and modulation of MMP-2 by peroxynitrite and glutathione. The results suggest that peroxynitrite can trigger MMP-2 activation through limited proteolytic cleavage, while glutathione can modulate enzymatic activity through redox modifications of critical cysteine residues [71]. It is probable that these post-translational mechanisms can directly influence the proteolytic activity of MMP-2 and, consequently, impact the processes involved in extracellular matrix remodeling.

In addition, other factors are capable of playing a significant role in the regulation of MMPs, affecting their expression and proteolytic activity [75]. ROS, including free radicals such as superoxide anion (O^2−^) and hydrogen peroxide (H_2_O_2_), are produced under various physiological and pathological conditions, including oxidative stress [76]. For example, Sawiki [77] demonstrated that the synergy between MMP inhibitors and ROS-dependent modifications in contractile proteins play an important role in protecting the heart against oxidative stress [77]. In addition, the author emphasized that the regulation of MMPs by ROS can occur in several ways, such as direct activation of MMPs, oxidation of specific cysteines in the active sites of MMPs, and regulation of the expression of MMPs through intracellular signaling pathways, such as the activation of transcription factors responsive to oxidative stress [77]. In this context, this imbalance can lead to an increase in ECM degradation, resulting in loss of structural integrity of cardiac tissue, adverse ventricular remodeling, and cardiac dysfunction.

## 4. Mechanisms Underlying the Role of MMPs in COVID-19

The expression of MMPs varies among COVID-19 patients and is correlated with the onset and progression of the disease. MMP-1 is highly secreted and can cleave fibrillar collagens of types I, II, and III. It plays a critical role in vascular remodeling and the development of related diseases. MMP-1 is unequivocally elevated in patients hospitalized with COVID-19 [11,62,68]. Both levels and enzymatic activity of MMP-1 were upregulated in the peripheral blood of patients with COVID-19, especially in severe/critical patients [11,68]. Furthermore, abundant levels of MMP-1 were identified in patients who developed early fibrotic changes identified on computed tomography. Therefore, this metalloproteinase is described as a potential peripheral blood biomarker related to idiopathic pulmonary fibrosis [11]. Overexpression of MMP-1 leads to the hyperactivation of MMP-1/PAR1 signaling, resulting in increased expression of VEGF receptor 2, reduced vascular endothelial cell function, and excessive recruitment and activation of inflammatory cells. However, high content levels in patients with COVID-19 are not exclusive to MMP-1 [78].

Alongside the significant elevation of MMP-1 observed in COVID-19 patients, there have been reports indicating that the SARS-CoV-2 spike protein can stimulate the expression of MMP-2 to a lesser extent [63,64], as well as MMP-3 [64]. MMP-2 is part of the gelatinases, initially named due to their ability to cleave denatured collagen. The regulation of MMP-2 activity is highly complex and involves post-translational processes that may go beyond the simple regulation of transcription or protein abundance [38]. Furthermore, although MMP-2 gene expression can be influenced by factors such as cytokines, tumor necrosis factor alpha (TNF-α), and growth factors, such as epidermal growth factor (EGF), it is important to point out that changes in MMP-2 expression and abundance do not always reflect biologically relevant alterations in proteolytic activity [79], since MMP-2 activation is a critical event that involves a series of complex post-translational events [80]. In the cardiovascular system, reversible and regulatory post-translational changes that impact Cys residues of different proteins were observed. Interestingly, MMP-2 was recently identified as a key enzyme involved in several cardiac conditions related to increased oxidative stress [81,82]. MMP-2 is the most abundant MMP in the heart, being ubiquitously expressed and intensely post-translationally regulated [71]. When MMP-2 is activated, specific proteins are degraded, such as troponin I [83], myosin light chain-1 [84], and α-actin. Therefore, it may be responsible for cardiac dysfunction in ischemia-perfusion injury [85].

Generally, decreased plasmatic levels of MMP-2 content in patients with COVID-19 represent a state of severe inflammation, similar to that seen in patients with sepsis [59]. However, a predisposition to increased MMP-2 content in the lungs has been observed in severe cases compared to mild cases of infection [14,62,86,87]. Increased levels of the MMP-2 protein observed in the cohort of D’Ávila-Mesquita et al. [67] in hypertensive individuals with COVID-19 are consistent with elevated levels of MMP-2 in bronchial cells infected with SARS-CoV-2 [59,67]. The authors [67] hypothesized that increased levels of mRNA MMP-2 in hypertensive patients with COVID-19 may result from an overactivated RAS. It has been observed that high levels of ANG II in COVID-19 are associated with endothelial injury and that this peptide increases MMP-2 protein expression levels [67]. The study also suggested that a reduction in MMP-2 content levels may result from systemic changes promoted by SARS-CoV-2, related to the cytokine storm and ACE2 imbalance [62,67]. More importantly, MMP-2 enzymatic activity has been linked to brain damage from this disease [59]. Elevated serum and cerebrospinal fluid (CSF) levels of MMP-2 enzymatic activity were identified in samples referring to individuals affected by COVID-19 with neurological sequelae [88].

Similar to MMP-2, MMP-3 is implicated in the onset of neurological damage in COVID-19 and is upregulated by the spike protein of SARS-CoV-2. MMP-3, a stromelysin, is potentially related to SARS-CoV-2 infection of host cells through cell fusion [65]. Although the spike protein can induce MMP-3 expression, MMP-3 is mainly synthesized by endothelial cells. Inflammatory cytokines stimulate the production and secretion of MMP-3 [65]. Shi et al. [65] showed a positive correlation between MMP-3 and inflammatory factors such as IL-1β and IL-6. In addition, a previous study reported more significant MMP-3 activity in tissues with large numbers of neutrophils, considered the main factor responsible for tissue damage [10]. Therefore, it has been suggested that MMP-3 activity aids in the migration of neutrophils from vessels to the extracellular space [64]. In any case, the increase in serum levels of MMP-3 levels of protein returned to normal in all 12 patients tested one month after admission. COVID-19 confirms an essential contribution of this protein during the initial phase of lung inflammation in the degradation of basement membranes [14,59,62]. Serum levels of MMP-3 protein content can serve as a valuable indicator of disease severity, as evidenced by the positive correlation between levels in patients with new coronavirus pneumonia and those without pneumonia.

A prior study observed that serum MMP-3 levels were significantly increased at admission of patients, and did not further increase after one week of hospitalization [10]. Subsequently, the MMP-3 serum levels returned to normal in all 12 patients tested one month after admission [10]. Furthermore, serum MMP-3 levels were related to the severity of lung injury in patients with COVID-19 [10,14,59,65]. In addition, because MMP-3 has previously been linked to traumatic brain injury, authors suggest that SARS-CoV-2 is a potentially neuroinvasive virus [68,88], which activates the mechanism to facilitate the migration of infected immune cells as a “Trojan Horse” to the brain parenchyma [64].

MMP-3 and MMP-7 also collaborate with the neutrophil infiltrate. The matrilysin MMP-7 is another immune cell mediator responsible for promoting neutrophil infiltration across the epithelial barrier, as it is responsible for an effective pro-inflammatory response [89]. MMP-7 is not able to cleave intact fibrillar collagens, being able to digest other ECM components. In addition, MMP-7 degrades the ECM deposited in the lung after injury [90]. According to Silva-Neto et al. [91], El-Din et al. [9], and Wu et al. [63], a significant increase in MMP-7 was identified in the lung tissue of those affected by COVID-19 when compared to those not infected [90]. In particular, patients who showed early pulmonary fibrotic changes on computed tomography were found to have significantly higher levels of MMP-7 proteolytic activity compared to those who did not exhibit such changes [92]. Therefore, serum MMP-7 levels were linked to early fibrotic changes in asymptomatic individuals with familial interstitial pneumonia [11,92]. Hence, circulating MMP-7 levels were described as a potential biomarker of COVID-19 severity [90,92] and potential peripheral blood biomarkers related to idiopathic pulmonary fibrosis [9,11,68,90,91,92]. As a result, MMP-7 can be used to aid in the identification of patients who will need mechanical ventilation [90,92]. According to Chun et al. [90], protein content in plasma levels of these MMPs was elevated according to the severity of COVID-19 and was significantly higher in the group admitted to the intensive care unit (ICU). This metalloproteinase is also associated with markers of pro-fibrotic factors, such as platelet-derived growth factor (PDGF) and transforming growth factor-β (TGF-β), as well as idiopathic pulmonary fibrosis [59,90]. MMP-7 is also related to the severity of COVID-19 in obese diabetics [10,18,68,93]. On the other hand, MMP-7 can serve as an indicator of the seriousness of post-COVID-19 lung injuries.

MMP-8, belongs to the collagenase family and has a chemotactic effect on neutrophils. Influenced by chemokines, neutrophils secrete MMP-8, facilitating the efflux of neutrophils through ECM degradation. In the same context, gelatinases A and B (MMP-2 and MMP-9) interfere through immune cells recruited from the peribronchovascular space in the alveoli, up to the modulation of chemokines. Silva Neto et al. [91] observed a relevant increase in the detection of MMP-8 in the lungs of people affected by COVID-19. Furthermore, another study reported that the plasmatic levels of protein content MMP-8 were elevated according to the severity of the infection [90]. However, Mohammadhosayni et al. [88] observed increased levels of MMP-3 in serum and CSF samples in individuals with COVID-19 and neurological syndrome [88], confirming the involvement of different structures through the action of the same MMP.

It is worth noting that the virus primarily targets alveolar epithelial cells, lymphocytes, and vascular endothelial cells. A marked increase in plasma levels of MMP-9, a gelatinase, was observed in patients with COVID-19 compared to healthy controls [63,66,87,90,94,95]. The spike protein from SARS-CoV-2 also slightly stimulates MMP-9 expression [64,66]. When the expression of MMP-9 is positively regulated, it promotes an inflammatory response, mediated by neutrophils, since MMP-9 is secreted by neutrophils and macrophages, with the migration of this enzyme from the endothelium to the sites of inflammation [66,90,93,95]. In addition, resident pulmonary epithelial cells only synthesize MMP-9 when subjected to various forms of injury [9,14,66,96]. In alveoli infected by COVID-19, there is an increase in inflammatory cells that release MMP-9, resulting in alveolar capillary destruction and consequent lung injury [9,13,59,91,94,95,97,98]. When the ECM is degraded, MMP-9 activates the derived growth factor and transforming growth factor beta 1 (TGF-β1) through binding with the receptor CD44 variant containing exon 6 (CD44v6) [97]. Furthermore, serum levels of MMP-9 are also related to the severity of COVID-19 [90,97,98]. In obese diabetics, high serum levels of MMP-9 were related to the severity of COVID-19 when associated with the pro-fibrotic markers PDGF and TGF-β [8,67,93]. The increased expression of MMP-9 also demonstrates an inflammatory response in the myocardium [12]. The literature suggests a pathogenic role of MMP-9 in systemic complications [95], beyond mortality [99]. On the other hand, MMP-9 is thought to possibly be implicated in the pathogenesis of neurological symptoms secondary to COVID-19 [67,88], just as they have also been identified as critical proteins for myeloid cell function [65]. Mohammadhosayni et al. [88] observed increased levels of MMP-9 in CSF and serum samples belonging to patients with COVID-19 associated with neurological syndrome compared to COVID-19 cases without neurological syndrome. However, the cellular origin and molecular pathways involved in MMP-9 release during COVID-19 remain unknown [97,99,100].

Although the MMPs described above have been implicated in COVID-19 infection, the involvement of other MMPs is less frequently reported. MMP-10, a neurodegeneration marker belonging to the stromelysin family, is elevated in positive cases of COVID-19 in individuals with cancer [12]. Remsik et al. [12] identified increased MMP-10 content in the CSF correlated with the degree of neurological dysfunction in this public. Thus, MMP-10 demonstrates interesting potential to be considered a prognostic biomarker in the future [12]. Further research is needed to elucidate how MMP-10 may impair neurological function following COVID-19 infection in cancer patients [12]. The cytokine storm from excess inflammation in the lung results in acute respiratory distress syndrome (ARDS), edema, and endothelial damage [8,86]. In the presence of these inflammatory mediators, there is an increase in the secretion of MMPs at the site of inflammation, generating a pathological remodeling of the pulmonary ECM. More specifically, as macrophages increase in the lungs, MMP-12 and MMP-28, which are secreted by macrophages and contribute to the pro-inflammatory process, become more prominent. MMP-12 plays a role in immune cell extravasation and migration to the brain [64,88]. With the help of other molecules, including cytokines, chemokines, and adhesion molecules, MMP-12 is potentially involved in developing neurological symptoms resulting from COVID-19 [64,87,88]. The spike protein found in SARS-CoV-2 can directly impact the function of the blood-brain barrier, providing a wider perspective on the neuropathological implications associated with COVID-19 [64]. The spike protein has also been reported to increase gene expression of MMP-12 [64]. Zerimech et al. [101] reported that subjects with fatal ARDS who were infected with COVID-19 had significantly elevated serum MMP-12 protein content during their ICU stay. The authors hypothesize that MMP-12 release by neutrophils and alveolar macrophages contributes to diffuse alveolar damage. These metalloproteases can degrade elastin fibers, primordial components of the interstitial matrix in the airways, and are essential for viscoelastic properties [101].

MMP-13, called collagenase, was also identified as participating in the alterations resulting from COVID-19. In severe cases of COVID-19, an increased expression of MMP-13 levels in epithelial cells of bronchoalveolar lavage fluid was evidenced compared to mild cases of this disease [101]. In addition, there is a trend towards increased levels of MMP-13 in severe cases compared to mild cases [24]. Silva-Neto et al. [91] also found a relevant increase in MMP-14 in lung tissue in patients affected by COVID-19 compared to non-infected individuals [91]. However, the mechanisms by which this modulation in levels occurs are still unclear in the literature.

Interestingly, MMPs are capable of modulating host tolerance to COVID-19 [65]. The activity of MMPs probably interferes with the effectiveness of therapeutic approaches directed at traditional pathogens. Therefore, this review encompasses established knowledge and insights regarding the mechanisms by which MMPs contribute to the transition from health to disease. Table S1 (Supplementary Material) summarizes all studies carried out on regulating MMPs and the tissues compromised by the COVID-19 infection.

## 5. SARS-CoV versus MERS-CoV

MERS-CoV (Middle East Respiratory Syndrome) is a virus that belongs to the genus Coronavirus. In most cases, symptoms of MERS-CoV infection appear between 2 to 14 days after exposure to the virus. In this sense, patients with mild MERS-CoV infections may experience symptoms such as low-grade fever, runny nose, malaise, dry cough, sore throat, and muscle aches. However, a complication called acute respiratory distress syndrome (ARDS) can develop in more severe cases. Complications are related to respiratory distress and pulmonary insufficiency [102]. Das et al. [103] observed that many patients who recovered from MERS-CoV infection developed pulmonary fibrosis in a relatively short period, with a progression ranging between 32–230 days [2]. In this way, the severity and duration of the disease were associated with the morbidity and extent of pulmonary fibrosis [103]. With symptoms similar to the aforementioned virus, the SARS-CoV coronavirus causes Severe Acute Respiratory Syndrome (SARS) [104,105], although it has relatively lower morbidity and mortality rates when compared to MERS-CoV [106].

These similarities are not limited to symptoms only, as both viruses can trigger profound inflammatory cytokine storms, with broad tissue tropism and intense interferon suppression [105,106]. However, there is a scarcity of information in the literature that associates the activity of metalloproteinases in pulmonary fibrosis caused by SARS-CoV and MERS-CoV in humans. In experimental studies with animals, Den Brand et al. [107] observed upregulation of expression levels of pro-inflammatory cytokines, chemokines, and MMP-4 in the bronchoalveolar lavage and in the respiratory tract of Rhesus monkeys [107]. Likewise, Sengupta et al. [60], in a previous analysis of the expression of MMP and TIMP in the central nervous system, after infection with the murine hepatitis virus (a member of the β-coronavirus genus, as well as SARS-CoV-2 and MERS-CoV), indicated that the increase in circulating levels of MMP-3 mRNA, MMP-12, and TIMP-1 are correlated with high viral replication [60]. However, it is important to highlight that there is a significant gap in the scientific literature of studies that specifically correlate the activity of matrix metalloproteinases (MMPs) during MERS-CoV and SARS-CoV infection. Although MMPs play a crucial role in regulating the immune response and in the pathogenesis of several diseases, including respiratory diseases, understanding of their specific involvement in these viral infections remains limited. More research is needed to investigate and elucidate the activity of MMPs during MERS-CoV and SARS-CoV infection, in order to gain a more comprehensive understanding of the mechanisms underlying these diseases and to develop potential therapeutic strategies.

## 6. Crosstalk between MMPs and Other Signaling Pathways

Several inflammatory diseases are related to the activation of the inflammasome pathway. This pathway is characterized by the presence of three main components, namely the protein of the Nod-like receptor (NLR) family, NLRP3, pro-caspase-1, and the apoptosis speck-like protein containing a CARD (ASC) adapter [104,105]. In turn, the activation of NLRP3 requires two signals. The first step of NLRP3 inflammasome activation is priming the cell with an NF-kβ activator, which leads to its own involvement [105]. The second signal involves a variety of activators, one of the main pathways including the P2X7 purinergic receptor. This receptor detects cellular ATP released after cell death, in order to induce potassium efflux and pannexin-1 recruitment. This occurs because pannexin-1 is a pore in the membrane that enables the delivery of patterns of extracellular pathogen-associated molecules (PAMPs) and patterns of danger-associated molecules (DAMPs) to the cytosol. NLRP3 expression occurs through myeloid cells, being regulated and activated in response to the stimulation of macrophages with PAMPs or DAMPs [106]. Furthermore, NLRP3 interacts with ASC and pro-caspase-1 to become effective. Then, after activation of the inflammasome assembly pathway, caspase-1 cleaves pro-IL-1β and pro-IL-18, and, finally, the biologically active forms IL-1β and IL-18 are secreted.

IL-1β is a prominent cytokine in inflammation, innate immune response, and fibrosis. It is produced by activated monocytes, macrophages, and dendritic cells, inducing the production of chemokines or cytokines, such as TNF-α and IL-6, in addition to proteases, such as MMPs. These MMPs are associated with neutrophil recruitment and fibroblast proliferation [107]. Since mature IL-1β is very potent, its production is tightly regulated in expression, transcription, and secretion [104]. MMPs are the main regulators of tissue and organ homeostasis [62,90,108]. Furthermore, they mediate tissue and organ damage when activity is pathologically elevated [9,10,11,12,13,14]. ECM degradation via MMPs can remove physical barriers, allowing cells to migrate and possibly invade adjacent tissues. The presence of MMPs in certain cells confirms that these cells can be sources of MMPs whose release occurs in the ECM. It also suggests the function of signaling cells and intracellular pathways [20].

In SARS-CoV-2 infection, inflammation also results in increased levels of pro-inflammatory cytokines, in addition to a range of other inflammatory markers and inflammatory transcription factors [109]. When inflammatory mediators are increased, they promote the activation of inflammatory cells responsible for releasing proteases, such as MMPs, whose post-translational activation can be catalyzed by other MMPs. In addition, oxygen radicals may also be released [109]. “Unbalanced” or excessive proteolytic activity results in imbalance of the MMP-TIMP complex and can lead to the activation of MMPs by other MMPs [91]. Activation of MMPs can activate TNF-α, and consequently deactivate AMPK. Increased MMP proteolytic activity, increased TNF-α levels, and reduced AMPK activity may synergistically and negatively impact metabolic pathways that are regulated by AMPK [8]. Furthermore, elevated levels of inflammation-regulated transcription factors have been described in the literature as being related to the cytokine storm in patients with severe cases of COVID-19 [109]. As a consequence of these changes, there is tissue injury [109]. Overall, the crosstalk between MMPs and other signaling pathways and molecules in COVID-19 is complex and still not fully understood. However, these interactions could provide potential therapeutic targets for the treatment of COVID-19 and its complications.

## 7. MMPs in Multi-System Physiological during COVID-19

Evidence suggests that when the immune response of the person affected by COVID-19 is exacerbated, several organs and tissues can be injured [88,101]. Changes in the immune system of individuals with COVID-19 were identified in CSF analysis, with antibodies present [88,101]. In such cases, these proteins can interact in a harmful way with the central nervous system [110]. In addition, an elevated number of monocytes was evidenced in CSF samples from those infected with COVID-19 with neurological symptoms and higher levels of MMPs-2, -3, -9, -10, and -12 [12,88]. There have been reports of strokes, acute inflammation, and demyelination of the central or peripheral nervous system [111,112]. By examining CSF samples from living subjects, the presence of neuroinflammation and gross immune response during acute COVID-19 was identified [111]. In these cases, the expression of regulatory genes by interferon in dendritic cells, associated with activated T cells and natural killer cells (NK) was observed in the CSF. Concomitantly, increases in IL-1 and IL-12 were not observed in blood plasma [111]. Even in the acute phase, different markers of monocyte activation and neuronal injury can be identified. In the subacute phase, critically ill patients present a decreased response to interferon and depletion of T cells in the CSF [113]. For instance, COVID-19 infection in the acute phase allows the appearance of neuropsychiatric effects. Mental confusion, stroke, and neuromuscular disorders may arise from neuroinflammation, coagulopathies, neuronal lesions, and possible viral infection in the central nervous system [112,114,115].

Cardiac tissue can also be severely compromised in COVID-19 [116,117,118]. In most cases, increased cardiometabolic demand associated with systemic infection and persistent hypoxia is a consequence of severe pneumonia or ARDS [118]. Another form of myocardial injury occurs through the interaction of SARS-CoV-2 with the ACE2 receptor, causing pulmonary, cardiac, and endothelial lesions [116,117,118]. Furthermore, elevated levels of MMP-9 have been linked to an inflammatory response in the myocardium, which is regularly associated with acute myocardial infarction [19].

In the lungs, COVID-19 activates neutrophils, and they release neutrophil extracellular traps (NETS), resulting in lung epithelial cell apoptosis and lung injury. It has been observed that the presence of neutrophils in the lungs of affected individuals can increase thrombosis, activating platelets [119]. In addition, the disease can cause changes such as hypoxemia, activation of the coagulation cascade, and destruction of endothelial cells, resulting in significant microvascular thrombosis [120]. The presence of ACE2 in the diaphragm of patients with COVID-19 has been reported [65]. Located predominantly in the muscle fiber membrane of the diaphragm, ACE2 is a way for SARS-CoV-2 to infect the muscle fibers of the diaphragm. Fibroblast growth factor signaling was also observed, representing the activation of fibrosis pathways [65]. MMPs participate in pulmonary immunity by facilitating the egress of inflammatory cells, modulating the activities of chemokines and cytokines, and activating defensins [121]. Furthermore, they contribute to the intercellular signaling in lung tissue. The consequence of this increase is the destruction of areas of lung tissue due to pathological remodeling of the ECM [14,62,63,86,87,101]. In dysregulation of MMP activity, there is the possible emergence and progression of different pathological conditions, including cancer, asthma, COPD, pulmonary emphysema, and pulmonary and hepatic fibrosis [122].

The alterations in MMPs in the plasma, serum, and peripheral blood mononuclear cells during COVID-19 reflect changes in the innate immune system and provide crucial information on inflammatory states. Plasma levels of MMP-1 were elevated in patients hospitalized with severe COVID-19 [11,62,68]. As for MMP-3 levels, increased serum levels during the course of this disease confirm the high contribution of MMP-3 in the initial phase, especially in lung inflammation and basement membrane degradation [14,62]. Therefore, an increase in MMP-3 serum levels on hospital admission was reported, characterizing the presence of an inflammatory state caused by COVID-19, with a subsequent reduction in MMP-3 levels after 1 month of cessation of systemic inflammation [10]. It is suggested that circulating levels of MMP-7 are related to idiopathic pulmonary fibrosis [9,11,63,68,90,91,92]. Plasma MMP-7 contents are increased in mechanically ventilated ICU patients [90], establishing a relationship between elevated levels of MMP-7 expression and the severity of respiratory compromise. Plasma levels of MMP-8 were also elevated according to the severity of disease symptoms [90]. Furthermore, plasma levels of MMP-9 were increased in those infected with COVID-19. Increased serum levels of MMP-9 have also been associated with the severity of COVID-19 [63,66,87,90,94,95]. The literature suggests that the pathological activity of MMP-9 is associated with systemic complications of the disease [94]. Other authors also found increased serum levels of MMP-12 during the ICU stay of critically ill patients with diffuse alveolar damage [101].

Regarding the digestive tract, a pathological process peculiar to patients without typical symptoms attributable to the disease is supported. In this case, the characterization was due to abdominal thrombosis [123]. MMP-9 has also been identified, with involvement in inflammatory modulation in intestinal pathological conditions, being upregulated by the increase in VEGF in intestinal disease [124]. Interestingly, in a study to associate MMP-9 activity with gastrointestinal changes resulting from COVID-19, the results suggested that MMP-9 is not involved in the pathogenic process related to the virus [124]. Data from the study showed possible involvement of the immunoglobulin CD147 in gastrointestinal infection by COVID-19, which may act in the presence of low expression levels of ACE2 [123]. Although CD147 induces MMP-9 expression [123], an increase in enzyme expression was not identified, even with high expression of CD147 [123].

**Figure 3:**
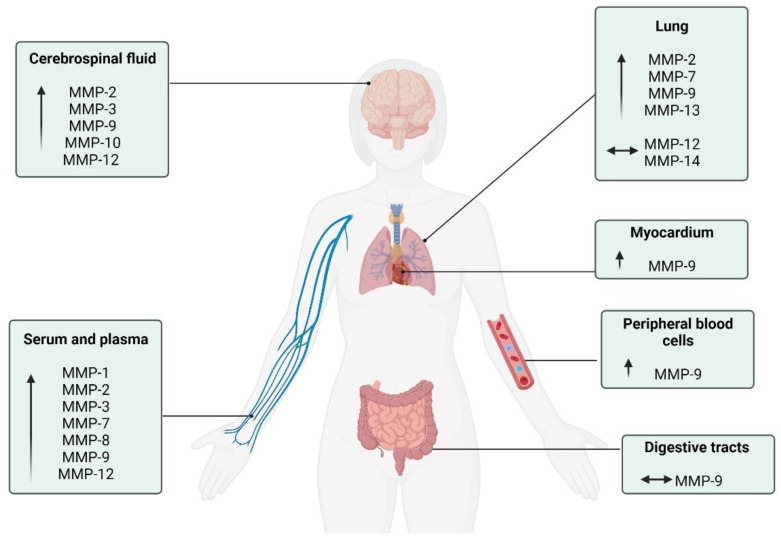
Schematic description of the MMPs activity in the different physiological systems of patients with COVID-19. ↑: increase compared to control individuals; ⟷ There was no change compared to other group.

Despite substantial progress in understanding the pleiotropic and multi-systemic actions of MMPs, the effects of COVID-19 on MMPs are mainly focused on blood circulation, neglecting the responses in other metabolic tissues. Future studies are necessary to elucidate the function of these enzymes in the skeletal muscle, bone, tendon, kidney, liver, pancreas, and adipose tissue, since these other tissues are also responsive to MMP modifications during chronic diseases [16,125]. Figure 3 summarizes the diverse MMPs in the different physiological systems.

## 8. Involvement of MMPs in Inflammation, Oxidative Stress, Circulation

### 8.1. MMPs and Inflammation in COVID-19

Inflammation is a central mechanism responsible for interacting with tissue-damaging stimuli resulting from pathogenic invaders or other means that cause damage. It aims to combat and remove the threat, heal the tissue, and restore homeostasis [126,127]. It is a process composed of several stages and several interconnected regulation methods, one of which is proteolysis, that involves matrix metalloproteinases [127,128].

MMPs can exhibit both pro-inflammatory and anti-inflammatory properties. They enable the recruitment and elimination of inflammatory cells by cleaving inflammatory mediators, generating a highly regulated inflammatory response [129,130]. MMPs can influence inflammation at several levels, including the regulation of the transmigration of inflammatory cells from the vasculature to the site of the inflamed tissue. These proteases also modulate the recruitment and influx of inflammatory cells to the site of inflammation, processing ECM elements, growth factors, cytokines, and chemokines [129,131].

Cytokines and chemokines are considered endogenous inflammatory mediators. They are responsible for inducing the migration of leukocytes to sites of injury or infection and activating cells to trigger an immune response [31,132]. MMPs can induce or suppress inflammation through direct proteolysis of inflammatory cytokines, thereby activating, inactivating, or antagonizing their function [31,132]. There are progressively more records that indicate the association between the immune response and the progression of COVID-19. Changes in immune cells, inflammatory markers, and the cytokine storm were related to the severity and evolution of the disease [31,133].

The severe inflammatory process is associated with disease severity and prognosis. Arshad et al. [134] analyzed two inflammatory markers, namely serum c-reactive protein (CRP), and lactate dehydrogenase (LDH). The authors conducted a study containing 283 patients positive for COVID-19 and ferritin, recording their efficiency as predictors of mortality [134]. In another study, Zeng et al. [135] observed in severe cases of COVID-19 a series of elevated inflammatory markers, such as IL-6, IL-8, IL-10, TNF-α, procalcitonin, ferritin, LDH, and high sensitivity CRP, as well as the relationship between CRP and high sensitivity lymphocytes [135]. The hyper-inflammatory response observed in COVID-19, manifesting as a cytokine storm, contributes to disease severity by increasing serum levels of IL-6, IL-8, TNF-α, and IL-1β. Elevated levels of these cytokines are associated with reduced chances of patient survival. There was no relevant decline in cytokines and inflammatory markers during illness [10,61,63,81,84,91,92,93,136]. In this context, an early reduction in inflammatory markers resulted in better outcomes [135]. Therefore, it is possible to recognize the critical role of the inflammatory process in the severity of COVID-19 and the participation of MMPs in this process.

### 8.2. MMPs and Oxidative Stress

Protein phosphorylation, activation of transcription factors, apoptosis, immunity, and differentiation depend on the adequate production and maintenance of reactive oxygen species (ROS) levels within cells, which must be carefully regulated to prevent oxidative damage [137]. When ROS levels increase, deleterious effects appear on prominent cellular structures such as proteins, lipids, and nucleic acids [138]. The imbalance between the production and accumulation of ROS in cells and tissues and the ability of the biological system to detoxify these reactive products results in oxidative stress (OS) [139].

OS can regulate the activity of MMPs. Specifically, increased levels of OS and peroxynitrite action can activate MMPs, particularly pro-MMP-2, even in self-inhibitory propeptide [72]. This ensures the maintenance of the same molecular weight as the active form (72kDa). In addition to the role of ECM remodeling, recent records show that MMP-2 also plays a relevant intracellular role, especially in response to OS [140,141]. Interestingly, the pathogenesis of COVID-19 appears to have been disturbed by OS [68]. The development of OS due to ROS and the depletion of antioxidant mechanisms play an important role in viral replication and the pathogenesis of subsequent virus-associated diseases [68]. In COVID-19 conditions, a common factor appears to be impaired redox homeostasis, which is responsible for the accumulation of ROS.

Furthermore, according to Martinez Mesa et al. [98], in one of the few studies published in the literature that relates OS biomarkers to the progress of COVID-19, ROS also produces other types of damage. These include protein damage, peptide chain fragmentation, electrical charge change, cross-linked reaction product aggregation, enzymatic inactivation, and susceptibility to proteolysis. Thus, these changes result in endothelial dysfunctions [98]. Considering that COVID-19 can increase OS and that OS can modulate the activity of MMPs, this may help to open avenues for studies involving the development of targeted strategies for effective potential therapies involving OS modulation.

### 8.3. MMPs and Circulatory Metabolism

Given the differences in both structure and function between veins and arteries, caution should be exercised when generalizing the effects of MMPs on veins to arteries. Nevertheless, MMPs likely contribute to vascular remodeling through various mechanisms, such as proteolysis of type I collagen (COL1), modulation of platelet-derived growth factor (PDGF) signaling, regulation of perivascular cells, and processing of vascular endothelial growth factor (VEGF) [130]. Some authors indicate that MMP-2 and MMP-9 reduce calcium influx in arteries and veins [142,143]. Despite this, MMP-2 expression is higher in vascular smooth muscle cells (VSMCs) from human saphenous veins than in those from human coronary arteries. In contrast, MMP-3, -10, -20, and -26 expressions are lower in saphenous veins than in coronary artery VSMCs [20].

Buzhdygan et al. [64] suggested that MMP-3 stimulates the migration of neutrophils from vessels to the extracellular space, contributing to tissue damage in COVID-19 infection. These findings underscore the importance of further studies investigating the differences in the expression and activity of MMPs in veins and arteries and in diseases that affect these vascular systems. Severe cases of COVID-19 have sepsis-like features [144], with MMP-2 and -9 considered potential biomarkers for septic patients [145,146]. Plasma levels of MMP-2 were reduced, while plasma levels of MMP-9 were increased in severe cases of the disease [67]. Figure 3 below schematically represents the increased MMPs and the respective suffering tissue.

### 8.4. The Severity of COVID-19 on MMPs Levels

According to the World Health Organization (WHO), the severity of the disease in patients affected by COVID-19 can be subdivided into four different stages, namely: Mild, Moderate (Pneumonia), Severe (Severe Pneumonia), and Critical (Respiratory Failure associated with Pneumonia). In this review, most of the authors included followed the WHO criteria for classifying the severity of illness by COVID-19 [147]. In mild disease, patients were asymptomatic or symptomatic, without evidence of viral pneumonia and/or hypoxia (SPO_2_ > 90%) [147]. To classify a patient as having a moderate case of the disease, they were required to present clinical signs of pneumonia (cough, accelerated breathing, dyspnea, and fever) but without evidence of the presence of severe pneumonia (SPO_2_ ≥ 90%). In the case of critically ill patients, clinical signs of severe pneumonia (high fever, dyspnea), extremely rapid breathing, around 30 breaths/minute, severe respiratory problems, and SPO_2_ < 90% were observed. Critically ill patients had all the characteristics of an extreme case but were intubated at some point during hospitalization. Among the main existing MMPs, during the COVID-19 pandemic, the ones that stood out were MMP-2 and MMP-9. In this perspective, authors such as Abers et al. [97], Ueland et al. [98], and Martinez-Mesa et al. [98] indicated a marked expression of MMP-9 in critically ill patients with respiratory failure, associating it with the risk of death. Furthermore, Abers et al. [97] mentioned that MMP-9 was associated with pulmonary fibrosis and a possible early indicator of respiratory failure [97]. Besides MMP-9, which has been shown to be associated with a high risk of mortality, others, such as MMP-1 [11,68,87,98,100], MMP-2 [67], MMP-8 [91], and MMP-13 [58,136] have also been correlated with disease severity and increased risk of death, especially during the acute phase of COVID-19.

The MMPs, as expected, were more expressed in the groups of severe and critical patients when compared to the other groups [11,58,59,81,82,84,85,90,136]. Metalloproteinase activity is closely linked to the inflammatory response and the activation of immune cells in the first line of defense, such as neutrophils. An increase in metalloproteinase activity, for instance, can indicate an inflammatory condition and the activation of immune cells, as well as the activation and exacerbation of pulmonary macrophages, which have been associated with high levels of MMP-7 and MMP-9 activity [9,14,59,63,90,91,94,95,97,98,100]. Another study pointed to the presence of four immune-related genes that are positively regulated in the face of covid-19 infection, namely, Toll-like receptor 6 (TLR6), SRC kinase-associated phosphoprotein 1 (SKAP1), lymphocyte activation gene 3 (LAG3), and MMP-9 [66]. This association can activate T cells and proteins fundamental to myeloid functions [66]. Therefore, one of the main differences between the moderate, severe, and critical stages is due to the exacerbated inflammatory response mediated by the immune system, by first-line defense cells.

However, our review identified several limiting factors that must be considered when interpreting the activity of MMPs and their possible correlations with the severity of COVID-19 patients. We emphasize the critical importance of conducting studies describing time points during data collection and rigorous statistical analyses. The lack of a consensual standard on how to report this information related to the expression of MMPs, impairs understanding of the behavior of these important molecules. Last but not least, most of the studies in this review did not analyze the respective inhibitors of metalloproteinases (TIMP) during COVID-19 infection, making it challenging to investigate the mechanisms of activation and inhibition.

## 9. The Influence of MMPs on Comorbidities

Understanding the role of MMPs in maintaining health is crucial for identifying tools to improve disease management. The dysregulation of these enzymes has been linked to numerous pathological conditions, such as inflammation, abnormal angiogenesis, microglial activation, tissue destruction, fibrosis, matrix weakening, and autoimmune diseases [15]. Of the 31 studies found, only 22 reported previous comorbidities in patients affected by COVID-19. The main illnesses described were diabetes mellitus (72.41%), cardiovascular diseases (62.06%), chronic lung diseases (37.93%), obesity (41.37%), autoimmune diseases (17.24%), kidney diseases (27.58%), liver diseases (10.34%), neurological diseases (3.44%), and gastrointestinal diseases (3.44%).

### 9.1. MMPS and Diabetes

Diabetes mellitus (DM) is a significant public health problem caused by unstable insulin secretion and/or insulin resistance, impairing carbohydrate, protein, and fat metabolism. The global prevalence of diabetes was 8.5% in 2014, and estimates suggest that the number of people affected will increase from 422 million to 642 million by 2040 [148,149]. Since the onset of the COVID-19 pandemic, individuals with diabetes have been classified as a high-risk population due to increased mortality rates and hospital admissions, and greater disease severity [150]. Due to the pathophysiology of the disease, in addition to affecting the immune system [151], diabetes is also capable of causing a pro-inflammatory state, with an exaggerated cytokine response [152].

MMP-1 was positively correlated in patients with diabetic retinopathy, and may act as a facilitator of angiogenesis [153]. In obese individuals, an increased concentration of MMP-2 has been linked to cardiovascular disease [154], as it is potentially induced in the adipose tissue of obese individuals and contributes to the increase in extracellular collagen [155]. Furthermore, attenuation of MMP-3 was observed in the adipose tissue of retinoic acid receptor orphan mice, which is related to increased adipogenesis and improved insulin sensitivity in a diet-induced obesity model [156].

According to some studies, hyperglycemia has been demonstrated to suppress the phagocytic capacity and increase MMP-9 expression [152]. Chronic activation of MMP-9 can result in the release of pro-fibrotic markers leading to increasing fibroblasts released, promoting fibrogenesis and pulmonary stiffening in diabetic patients, making them more vulnerable to infections, for example COVID-19 [86,157]. Meanwhile, some studies have found that diabetic patients exhibit lower levels of MMP-2 compared to control groups [158] and an inverse correlation between serum levels of MMP-9 and body mass index [14]. From another perspective, the attenuation of MMP-13 is positively correlated with the treatment of diabetic neuropathy [98]. MMP-13 is expressed in intact skin and physiological conditions at relatively low levels, increasing in the acute period of skin injury [158,159]. Glucose has glucose-dependent neurotoxic effects that can be explained by the high formation of ROS in the skin. ROS stimulate the expression of MMP-13 and promotes the susceptibility of the epidermis to mechanical stress [159]. At the same time, the intensification of MMP-11 expression is benign against DM [160], as it plays a systemic role in the maintenance of glucose and lipid homeostasis, increasing insulin sensitivity.

### 9.2. MMPs and Cardiovascular Diseases

Arterial hypertension is the most frequent preventable cause correlated with cardiovascular morbidity and mortality and is considered a truly global epidemic, with estimates of approximately 1.3 billion people affected [161,162]. The association between hypertension and COVID-19 was reported in early studies and identified as a global phenomenon [163]. One possible explanation for this is the relationship between the COVID-19 virus and the renin-angiotensin system (RAS) [139]. This is because the virus can inhibit the expression of ACE2, leading to the uncontrolled activity of angiotensin 2, which has vasoconstrictor effects and is associated with endothelial dysfunction and inflammation [139]. MMP-2 is related to hypertension because it can degrade a series of myofilament proteins correlated with contractility, decreasing myofilament sensitivity to Ca^2+^, and triggering contractile dysfunction [164]. In addition, studies demonstrate that increased MMP-2 activity may contribute to eutrophic and hypertrophic remodeling because MMP-2 can participate in the development of vascular tone and induce hypertension-induced proteolysis, attenuating vasodilator signaling [165,166]. Of relevance to the current context, a recent study [67] demonstrated that plasma levels of MMP-2 were increased in hypertensive patients with COVID-19 compared to normotensive patients, although lower than those observed in non-hypertensive patients affected by COVID-19.

MMP-9 has a direct relationship with this arterial remodeling process, generating an increase in pressure due to the pathological distensibility of the vessels. This condition causes the cardiac and vascular tissue to present an extra compensatory remodeling, which generates cardiovascular complications [167]. Elevated levels of MMP-9 in hypertensive individuals have been correlated with arterial stiffness, as well as the development of early-stage hypertension itself, causing collagen degradation and arterial deterioration [168]. Although there is evidence linking MMP-9 to left ventricular hypertrophy, it has been reported to have a more significant impact on vascular remodeling and tone than on cardiac remodeling [169]. Therefore, the activity of MMPs can be enhanced during arterial hypertension, resulting in increased remodeling and continuous degradation of endothelial extracellular matrix components [168].

### 9.3. MMPs and Obesity

Obesity is defined as a disorder characterized by excessive and harmful accumulation of body fat, listed as subgroup E66 under the category of “endocrine, nutritional, and metabolic disorders,” a condition related to several serious consequences, including an increased risk of death, accelerated aging, and morbidity in individuals. In addition, obesity has been regarded as a common global pandemic disease, which has nearly tripled since 1975, according to the World Health Organization (WHO). The COVID-19 pandemic highlighted the vulnerability of the obese population, as excess body fat tissue can increase susceptibility to the virus [170]. MMPs have been implicated in the pathophysiology of various cardiometabolic and inflammatory disorders, and their role in disease progression makes them a relevant research target [171].

Divergent findings have been reported regarding the levels of specific MMPs in obese individuals., For example, elevated levels of MMP-1 were observed by Boumiza et al. [172], while another study revealed elevated levels of MMP-1, but not of MMP-2, MMP-3, and MMP-9 [172]. The study by Ozen et al. [173] reported marked levels of both MPP-1 and MMP-2 in obese subjects when compared to non-obese subjects. The hypothesis is that the inflammation perceived in obesity can stimulate the production of MMP-1 and MMP-2, which can generate adipose tissue remodeling [173,174]. On the other hand, the study by Belo et al. [174] demonstrated increased MMP-2 activity in the plasma of obese/hypertensive children and adolescents and reported that adiponectin levels were negatively associated with circulating levels of MMP-2, therefore making the individual vulnerable to cardiovascular diseases, especially in obese children and adolescents [174].

MMP-3 was associated with inflammatory responses during adipose tissue prolongation and increased adipose tissue levels, suggesting that MMP-3 plays a role in adipogenesis [175]. In contrast, the study by Wu et al. [63] demonstrated that MMP-3 expression in adipose progenitor cells plays different roles depending on sex. For example, attenuation of MMP-3 in female mice with high-fat diet-induced obesity was followed by increased fat cells. In contrast, there was an increase in MMP-3 activity and expression in obese male mice, accompanied by a reduction in fat cells [176]. However, little is known about the actual positive regulatory processes of MMP-3 expression in adipose and visceral tissues. There are specific differences regarding evidence linking serum levels of MMP-9. Some studies do not demonstrate significant differences [172,177,178] while others report attenuated levels of MMP-9 in obese individuals or those with metabolic syndrome [179].

Meanwhile, other studies point to increased MMP-9 expression in obese individuals [180,181,182]. The possible explanation for these discrepancies in the results could be due to methodological heterogeneity, such as differences in age, sex, and the samples evaluated. In addition, an interesting study even indicated that adipose tissue may not be the main contributor to circulating levels of MMP-9 [183]. From another perspective, the study by Li et al. [184] showed that MMP-14 can trigger processes that produce endotrophin, an adipokine that plays a crucial role in the development of fibrosis and inflammation. On the other hand, the study reported that MMP-14 has the opposite role during the early stage of obesity: digesting collagen to prevent overaccumulation of extracellular matrix in adipose tissue contributes to the healthy growth of fat pockets [184].

### 9.4. MMPs and Lung Disease

Although MMPs are produced by different lung cells, the precise association between certain MMPs and adjacent cells, their extracellular origin, and the limited availability of highly selective antibodies poses a challenge in identifying their specific localization within the lung [19,185]. However, macrophages appear to be the primary source of MMP production in the lungs, although other lung cells (epithelial and endothelial cells, for example) also participate in this process [186]. In the context of the COVID-19 pandemic, recent studies suggest that COVID-19 infects type II pneumocytes, leading to rapid replication of the virus and intense secretion of pro-inflammatory cytokines and chemokines, among other cells that participate in these inflammatory pathways. This rain of cytokines, specifically in the lung, can lead to ARDS, epithelial damage, and pulmonary edema [187].

Because ARDS is the main condition triggered by COVID-19, it is worth mentioning that its pathophysiology is marked by progressive arterial hypoxemia, respiratory failure, dyspnea, and increased work of breathing [187,188]. It is also closely related to lung injury, which may contribute not only to the formation of edema and inflammatory mediators but also to damage to the endothelium and epithelium caused by neutrophils [187,188], including cell death in these tissues, allowing the increase in the permeability of the alveolar-capillary barrier. Clinical data have demonstrated correlations with marked levels of MMPs in moderate to severe patients with ARDS and COVID-19-promoted lung injury. Among the MMPs involved in worsening ARDS are: MMP-1 [11,62,68], MMP-2 [63,67,88], MMP-3 [10,62,65], MMP-7 [9,11,63,90,92,93], MMP-8 [91], MMP-9 [9,10,13,14,59,62,63,81,82,87,88,90,91,92,150], MMP-10 [12], MMP-12 [88], and MMP -13 [59,136].

Several cytokines can be cleaved by MMPs, which generate activation, inhibition, or changes in bioavailability, contributing to a greater inflammatory profile in the injured lung [188]. Although studies support the role of MMPs in activating soluble chemokines, evidence demonstrates a clear and direct role in the degradation of the endothelial and epithelial barrier [189]. For example, MMP-2 and MMP-9 can degrade type 4 collagen, a fundamental component of basement membranes [190]. Furthermore, a recent study indicated a significant correlation between increased inflammation and growth factor signaling with elevated levels of MMP-3 [191]. MMP-7, on the other hand, participates in the positive regulation of migration and the accentuation of lesion closure [192]. Alternatively, the proteolytic cleavage of MMP-9 into IL-8, IL-1β, and CXCL6, which can strongly increase leukocyte recruitment to injured heart and lung tissue [193,194], is correlated with decreased injury repair [195]. In summary, MMPs can control cellular functioning by regulating several cytokines that play a role in the evolution of the response to inflammation in the lesion.

## 10. Time Course of MMPs Activity during COVID-19

Inflammatory cytokines exert critical effects in inflammatory diseases. They can be used as biomarkers, indicating or monitoring pathological progression. Furthermore, they are closely related to the activity of MMPs, considered biomarkers of progression in COVID-19 [196]. Studies on MMPs in people affected by COVID-19 at different stages and disease progression have brought compelling evidence. A body of studies recorded increased MMP-7 and MMP-9 activity in blood circulation when patients were admitted to hospital after being infected with this disease, characterizing the initial phase of the infection. This process is associated with lung damage, the inflammatory response in the myocardium, and neurological symptoms related to the initial inflammatory stage of the disease [9,13,14,59,68,88,94,95,97,98,99,100]. Parra et al. [62] also observed high levels of MMP-1 and MMP-3 in individuals positive for COVID-19 who were in the hospital emergency room, whether or not they were eligible for hospitalization [62].

Other authors indicated an increase in MMPs-1, -2, -7, -9, and -13 in blood circulation during hospitalization, referring to the persistent infectious state. These high levels resulted in pulmonary fibrotic changes, endothelial lesions, brain damage with neurological symptoms, and inflammatory response in the myocardium [11,59,63,68,88,90,92,98,100]. Petito et al. [94] also found increased levels of MMP-9 in individuals during COVID-19 infection but did not discriminate the period of illness [94]. Findings about elevated levels of MMP-9 deserve special attention, as they are also related to lung damage [9,14,59,91,94,97,98,100]. On the other hand, Silva Neto et al. [91] recorded an increase in MMP-8 in the blood plasma of moderate, severe, and critical COVID-19 patients at hospital admission and within 6 days of admission. This increase was also related in the onset of neurological sequelae [91]. Between 2 and 6 months after hospital discharge, higher levels of MMP-7 proteolytic activity were related to worse pulmonary diffusion, whereas elevated levels of MMP-1 proteolytic activity were linked to worse pulmonary diffusion and early fibrotic changes [99]. Recently, Kumar et al. [197] observed elevated levels of MMPs in children requiring intensive care unit admission compared to children not needing intensive care, reinforcing the idea that the hospitalization process can accentuate the increase in MMP activity.

**Figure 4:**
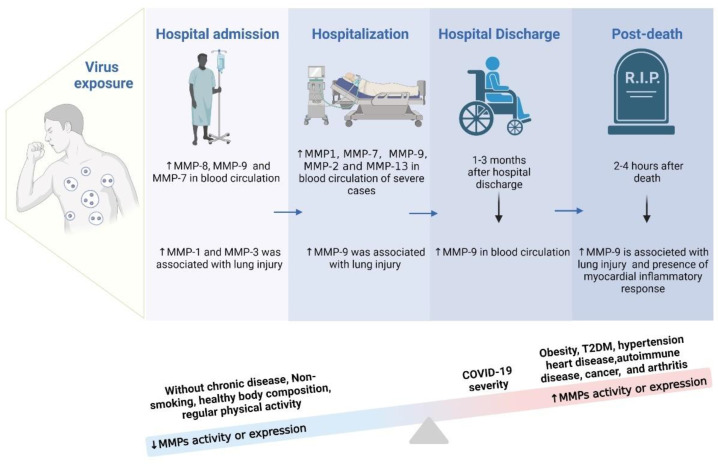
Identification of the most common MMPs during the hospitalization course time.

Finally, the records in the literature corroborate that MMP-9 expression remained altered in the post-mortem of COVID-19. Such findings are compatible with inflammation in the myocardium and with lung damage [13,14]. However, an important point is that the increase in MMP levels is also related to pre-existing health conditions and quality of life. In general, individuals who did not have chronic diseases, did not smoke, had good body composition, and practiced regular physical exercise did not develop the most severe stages of the disease [140,141,198,199]. This can be explained by the fact that these individuals did not present factors related to pre-existing systemic inflammation. Considering that COVID-19 is an inflammatory disease and that the increase in MMP levels results in inflammation of different tissues, individuals who presented a more precarious state of health had a worse prognosis [140,144,198,199]. In short, individuals with obesity, T2D, hypertensive, heart disease, carriers of autoimmune diseases, individuals with cancer, and those with arthritis had higher levels of MMP activity when they were infected by and developed COVID-19. Consequently, a pre-existing inflammatory state led to a worse prognosis. Figure 4 shows a schematic representation of the increased MMPs in different periods after the COVID-19 infection, including factors related to greater or lesser activation of MMPs in people affected by COVID-19.

There is no consensus in the literature about the exact period in which MMPs remain altered in each stage of the disease, considering the different severities. However, Syed et al. [68] observed a significant increase in the expression levels of MMPs in patients hospitalized for COVID-19 compared to individuals with mild/moderate infection or healthy individuals. The study identified high levels of MMP-1 proteolytic activity in the peripheral blood of individuals affected by COVID-19, particularly those with severe and critical illness [68]. However, there have been no reports on the duration of increase in MMP-1 proteolytic activity considering baseline levels [68].

Gelzo et al. [10] identified that MMP-3 gene expression was increased in individuals infected with COVID-19 compared to controls. Although serum levels of MMP-3 increased after one week of hospitalization, expression levels of MMP-9 did not change with advancing disease stages, despite being higher in those affected by COVID-19. Furthermore, MMP-3 mRNA levels returned to baseline values in 12 patients after one month of hospitalization [10]. Chavez-Galan et al. [92] identified elevated mRNA levels of MMP-7 in the lung tissue of patients who developed early fibrotic changes, but did not report how long they remained high. Chun et al. [90] also observed high MMP-7 levels in individuals diagnosed with severe COVID-19 in the acute phase of the infection, without recording further data related to the time of MMP-7 modulation. These findings highlight the importance of longitudinal assessment of MMP-7 levels in patients with COVID-19, in order to better understand its role in the progression of pulmonary fibrosis and the development of serious complications.

## 11. Therapeutic Approaches and Treatments That Can Modulate MMPs: Insights and Perspectives

### 11.1. Potential Strategies for the Normalization of MMPs

There is a significant global need to discover sophisticated therapeutic strategies that can repair and alleviate the damage caused by pathogens, especially in patients affected by COVID-19 [200]. Strategies capable of reducing the inflammatory process will benefit the population affected by COVID-19, including those who have recovered from the infection. Physical exercise is a stressor that causes inflammation during and after its practice [201]. The increased expression of pro-inflammatory cytokines appears critical for long-term adaptive responses to exercise. Interestingly, regular exercise can be adopted as a long-term anti-inflammatory therapy once the acute inflammatory period has passed [202].

Furthermore, there is also evidence-based drug support for the pharmacological treatment of the inflammatory process in COVID-19. Colchicine is one of the anti-inflammatory resources used in this infection. A study with a sample of 4488 participants with mild and moderate COVID-19 randomized 2235 individuals into the group that used Colchicine and 2253 people into the placebo group [203]. During the first three days and once daily for the remaining 27 days of the trial, colchicine was administered at 0.5 mg. Before randomization, the mean duration of symptoms was 5.4 ± 4.4 days. The approximate age in the colchicine group was 53 years, and in the placebo group 54 years. A sensitivity analysis performed on COVID-19-positive patients showed that the primary efficacy endpoint occurred in 96 of 2075 patients in the colchicine group (5%) and 126 of 2084 (6%) patients in the placebo group. Therefore, this study clarifies that colchicine can be administered to patients on an outpatient basis, with or without confirmation of the disease, aiming to contain clinical deterioration and future hospitalization [204]. This drug can inhibit the release of pro-inflammatory cytokines induced by the inflammasome secondary to viral infection [204], as well as neutrophil chemotaxis and aggregation [203]. Therefore, it can be used in the treatment of different inflammatory diseases.

Evidence shows that anakinra can inhibit inflammation and improve the prognosis in patients with COVID-19 [150,205]. Because anakinra is a type of human recombinant IL-1 receptor antagonist, its use was recommended based on increased IL-1βn patients with COVID-19 [150,205]. Furthermore, the inhibition of specific interleukins has also shown benefits in the treatment of COVID-19. Evidence also supports the early use of tocilizumab, a monoclonal IL-6 antibody. The use of this antibody has been especially attributed to helping critical patients [206,207].

Another study tested the beneficial effect of fluvoxamine on the inflammation characteristic of COVID-19 [208]. Fluvoxamine is a selective serotonin reuptake inhibitor used in treating patients with mild to moderate COVID-19, due to its inflammatory, antiviral, and inhibitory platelet activation properties. It acts through strong stimulation of the alpha-1 receptor in the endoplasmic reticulum, mitigating the inflammatory process that responds to infection and resulting in cytokine-regulated production [209]. A clinical trial was performed with 80 outpatients in the intervention group (100 mg, three times a day) and 72 placebo patients. The mean age was 46 years, and comorbidities were included, with 54% of patients being obese. Clinical deterioration within 15 days after randomization was the main efficacy endpoint, which was found in 0% of patients in the fluvoxamine group and in 6 (9%) in the placebo group [208].

Another possibility to reduce the inflammation present in COVID-19 is through Janus kinase (JAK) inhibitors, responsible for blocking the JAK signal transducer and the transcription-activating pathway (STAT) [210]. JAK-STAT is a cytokine-activating pathway involved in several relevant biological processes, such as cell proliferation, differentiation, apoptosis, and immunomodulation. JAK inhibitors can control immune activation and cell inflammation, inhibiting the cytokine storm [210]. 8 JAK inhibitors may decrease lung inflammation resulting from COVID-19 infection in rhesus macaques [210,211]. Other reports in the literature point to the possible use of other inhibitors in the treatment of COVID-19, including ruxolitinib [212], baricitinib [213], and bruton kinase [214].

Glucocorticoids are also used to combat COVID-19 inflammation due to their potent anti-inflammatory action. However, their use is not encouraged in the early stages of COVID-19 infection as they cause suppression of the immune system [215]. In this context, a large open-label, multicenter, randomized trial was conducted in the United Kingdom (UK), including patients with suspected or laboratory-confirmed COVID-19 [216]. The sample consisted of 2104 patients, divided between the group that received 6mg of dexamethasone once a day for ten days and 4321 patients that received only the usual care. At the end of the study, it was observed that dexamethasone reduced mortality in 28 days in patients with symptoms longer than seven days. Therefore, the regimen was put into clinical use in the UK [216].

On the other hand, using non-steroidal anti-inflammatory drugs (NSAIDs) remains controversial in treating COVID-19, as little data are available on the subject [217,218,219,220,221,222,223,224]. Several important advances in the treatment of COVID-19 have been identified and implemented since the disease evolved into a pandemic, making it possible to treat those infected and preventing the deterioration of the clinical picture.

### 11.2. Physical Exercise and Diet as Factors That Modulate MMPs in COVID-19

Physical exercise benefits health status and can be a complementary treatment for chronic diseases, as evidenced by numerous studies [225,226,227,228,229,230,231]. Regular exercise increases the ability of lymphoid cells to produce a humoral or cellular immune response when stimulated by an antigen. Studies suggest that COVID-19 severity is associated with the health status before infection [153]. Consequently, lifelong exercisers present a preserved immune response to exercise, indicating that they are better prepared to respond to new immune challenges. Thus, the enhanced anti-inflammatory responses inherent to proper exercise doses could mitigate COVID-19 complications [206,212].

Nevertheless, it is necessary to perform studies investigating the influence of different physical exercise modalities on MMP activity in various physiological systems of COVID-19 patients. Additionally, we suggest that acute training variables (intensity, duration, volume, frequency, and interval) be controlled, addressed, and successfully integrated to create an effective intervention, regardless of the exercise types. These aspects can be valuable for understanding the beneficial exercise effects, particulars, and restraints, and providing significant evidence for therapeutic approaches, besides aiding understanding of the potential mechanisms involved.

In addition to physical exercise, diet plays a fundamental role in the immune system [232,233]. Studies have shown that patients with COVID-19 and inadequate levels of micronutrients present more extended periods of hospitalization. Furthermore, most of those hospitalized with COVID-19 had a significant deficiency of at least one nutrient [228,234]. Several dietary compounds can exert pleiotropic effects on different targets, helping to relieve symptoms and promoting physical and psychological well-being through independent and synergistic mechanisms [232]. The purpose of nutritional interventions as a strategy to combat inflammatory properties will remain important until the long-term effects of COVID-19 are fully understood. Dietary components, such as vitamins C and D, omega-3 fatty acids, and zinc, have been shown to have anti-inflammatory effects [235,236,237]. Last but not least, studies are needed that investigate the role of diet in the modulation of MMPs post-COVID-19.

## 12. Conclusions

The studies included in this review highlight the potential of metalloproteinases (MMPs) as prognostic markers in assessing the severity of COVID-19, revealing that the infection stimulates mechanisms that activate MMPs due to the loss of homeostasis of the immune system. The activation of MMPs can occur through the secretion of these enzymes by immune cells, such as neutrophils and monocytes. The result of this activation involves negative impacts on tissue remodeling, inflammation, and oxidative stress associated with the disease, in addition to an association with disease severity and length of hospital stay. However, it is essential to recognize the limitations of the low specificity of MMPs for an accurate diagnosis.

MMPs are enzymes involved in several physiological and pathological pathways, which makes them less specific to uniquely identify the presence or severity of COVID-19. Other factors, such as the presence of chronic diseases or comorbidities, may influence MMP levels in the context of SARS-CoV-2 infection. In this sense, continuous investigation of the role of MMPs in COVID-19 and the validation of these findings in future studies is recommended. This information is essential both to advance understanding of the pathological mechanisms and to improve individualized assessments under the guidance of health professionals, as well as assisting in risk stratification, therapeutic decision-making, and the development of targeted strategies to mitigate the impacts of the disease.

## Data Availability

Not applicable.

## Supplementary Materials

The following supporting information can be downloaded at: https://www.mdpi.com/article/10.3390/biology12060843/s1, Table S1: Summary of all studies carried out on regulating MMPs and the tissues compromised by the COVID-19 infection.
