# Supplementary material for: Involvement of Matrix Metalloproteinases in COVID-19: Molecular Targets, Mechanisms, and Insights for Therapeutic Interventions
Source: Biology (Basel). 2023 Jun 10;12(6):843. doi: 10.3390/biology12060843 (PMC10295079; doi:10.3390/biology12060843)
Supplement: Supplementary file 1 [file biology-12-00843-s001.zip › biology-2397280-supplementary.pdf]

Table S1. Summary of all studies carried out on regulating MMPs and the tissues compromised by the COVID-19 infection

Authors	Study design	Sample characteristics	Age/ Gender	Disease Severity	Tissue	Tissue collect time point	Analysis Methodology	MMPs	Previous comorbities	Outcomes	Link- paper
ABERS et al. 2021	Cohort	Hospitalized patients with confirmed COVID-19 (n= 175), and control group (n= 43 healthy volunteers)	Hospitalized patients: x̄ age 60 years, males: n= 132, female: n= 40; control group: x̄ age 44,9 years, males: n= 23, females: n= 19	Critical, severe, mild or moderate	Serum or plasma	Hospital admission until 45 hospitalized days	Magnetic bead-based Multiplex assay (Bio-Plex 3D)	↑ MMP-9 compared with the control group	Hypertension, diabetes, malignancy, and autoimmune disease, heart disease, congestive heart failure, obesity (BMI >30), hyperlipidemia, chronic respiratory disease, chronic kidney disease, chronic liver disease, gastrointestinal illness	↑ MMP-9 in severe and critical COVID-19 patients. MMP-9 showed significant activity in neutrophil activation, which was associated with risk of death	https://www.ncbi.nlm.nih.gov/pmc/articles/PMC7821609/pdf/jciinsight-6-144455.pdf
PETITO et al. 2021	Case-controlled	Hospitalized patients with COVID-19 (n=36), and age- and sex-matched healthy controls (n=31)	COVID-19 group: x̄ age 70,6 years, males: n= 20, females: n= 16; control group: x̄ age 65 years, males: n= 12, females: n= 19	Admitted to the ICU (n= 6), required mechanical ventilation (n= 14), endotracheal intubation (n=9),	Plasma	During ICU hospitalization: 3-4 days after the last positive nasopharyngeal swab sample. After hospitalized: 2-3 months after	Enzyme-linked immunosorbent assay	↑ MMP-9 compared with the control group	Hypertension, Diabetes, obesity, smoking, atrial fibrillation, cirrhosis, kidney failure, stroke, peripheral artery disease	↑ MMP-9 in the plasma of patients with COVID-19. No significant differences were found for any of the MMP-9-related biomarkers between ICU and non-ICU patients	https://pubmed.ncbi.nlm.nih.gov/33280009/

				non-ICU (n=5)		hospital discharge, made two negative swab samples					
BUZHDYGAN et al. 2020	Experimental	SARS-CoV-2 subunit 1 (n=10 nanomer [nM]), SARS-CoV-2 Subunit 2 (n= 10nM), SARS-CoV-2 (n=10nM)	--	--	Human brain microvascular endothelial tissue	4 and 24 hours	qRT-PCR	RBD 4 H and 24 H: ↑MMP-2, ↑MMP-3, ↑MMP-9 and ↑MMP-12. Subunit-1 4 H: ↑MMP-3 ↑MMP-12. Subunit-1 24 h: ↑MMP-3, ↑MMP-9 and ↑MMP-12. Subunit-2 4H: ↑MMP-3 and ↑MMP-12. Subunit-2 24H: ↑MMP-2, ↑MMP-3, ↑MMP-9 and ↑MMP-12	--	SARS-CoV-2 spike protein can lead ↑ of MMP expression in brain endothelial cells, which supports a very specific pro-inflammatory response and a decreased brain-blood barrier resistance	https://www.ncbi.nlm.nih.gov/pmc/articles/PMC7547916/
UELAND et al. 2020	Letter to the editor	Hospitalized patients with COVID-19 (n=39), without respiratory failure (n=18), and respiratory failure (n=21)	Without respiratory failure: $\bar{x}$ age 60 years, males: n= 11, females: n= 7; respiratory failure: $\bar{x}$ age 61 years, males: n= 18, females: n= 3	Severe	Plasma	Day 0-2, day 3-5, and 7-10	Enzyme Immunoassays	↑ MMP-9 compared without respiratory failure	Chronic cardiac disease, chronic lung disease, chronic kidney disease, obesity and diabetes	MMP-9, sTNFR1 were consistently associated with respiratory failure. MMP-9 and GDF-15 were related to pulmonary fibrosis, suggesting that MMP-9 could be an early indicator of respiratory failure	https://pubmed.ncbi.nlm.nih.gov/32603675/

ADAS et al. 2021	Prospective double-controlled trial	Group 1/control (n=10): moderate condition; group 2/control (n=10): critical ill, intubated and conventional treatment in ICU; group 3/experiment (n=10): critical ill, intubated and followed-up	$\bar{x}$ age 56 years, males: n= 19, females: n= 11	Moderate and critical clinical cases	Mesenchymal stem cells	Day 0, day 3, day 6 in the hospital	Luminex Assays Human	↑ MMP-9 and ↑ MMP-13 in patients taken into ICU compared to those with infection clinic; ↓ in add-on, MSC transplanted critical cases	--	↑ MMP-9 and ↑ MMP-13 levels in acute lung injury released inflammation and degradation. These MMPs were ↓ in add-on MSC transplanted critical cases. It suggests that the immunomodulatory effect of MSCs was involved in the regulation of MMP levels	https://www.ncbi.nlm.nih.gov/pmc/articles/PMC8243094/
MOHAMMAD HOSAYNI et al. 2021	Experimental	Neurologic syndrome (NS) patients hospitalized ICU With COVID-19 (n=20); NS (n=10), and without NS (n=10; sex and age-matched)	NS: $\bar{x}$ age 61,21 years, males: n= 5, females: n= 5; without NS: $\bar{x}$ age 59,51 years, males: n= 5, females: n= 5	Severe and critical (inferred by internalization patient level)	Cerebrospinal fluid (CSF) and serum	--	ELISA	MMP-2(↔ Serum levels for both groups; ↑ CSF levels in NS) ,MMP-3(↑ Serum and CSF levels in NS and Without NS), MMP-7 (↔ Serum and CFS levels for both groups) ,MMP-8 (↔ Serum levels for both groups), MMP-9(↑ Serum and CFS Levels for both groups) AND MMP-12 (↑↑ Serum levels in both groups)	--	High levels of TNF-α can lead an ↑ expression of MMP-2, MMP-3, MMP-9 and MMP-12 from monocytes stimulation	https://www.ncbi.nlm.nih.gov/pmc/articles/PMC8367754/pdf/main.pdf

D'ÁVILA-MESQUITA et al. 2021	Experimental	Patients hospitalized ICU, with COVID-19 (n= 53), control group with healthy individuals (n=29), COVID-19 HPT group (n=29), HPT control group (n= 14), COVID-19 non HPT group (n= 24), control non HPT group (n=15), COVID-19 obese group (n=27), control obese group (n=13), COVID-19 non obese group (n=26), control non obese (n=16)	COVID-19: $\bar{x}$ age 59,54 years, males: n= 36, females: n= 17; control group: $\bar{x}$ age 57,8 years, males: n= 17, females: n= 12	Severe	Plasma	Within 48H of the ICU admission	Gelatin zymography	MMP-2(↑ in COVID-19 HPT group, but ↓ when compared with control group. ↔ in COVID-19 Obese group when compared with control group) MMP-9 (↑ in COVID-19 both groups compared to control group)	Diabetes, hypertension, and obesity	↑ MMP-2 levels, which means an overactivated renin-angiotensin system, albeit still downregulated in general. MMP-2 was correlated with mortality in COVID-19. ↑ MMP-9 levels in severe cases	https://www.ncbi.nlm.nih.gov/pmc/articles/PMC8376652/
HARTMANN et al. 2021	Case-controlled	Mainly male, hypertensive, diabetic, with a history of coronary artery disease. Symptomatic people who died from COVID-19 (n=6) were compared to a control group (n=11)	COVID-19 group: Md age 74 years, control group: Md age 73 years	All were admitted to ICU and developed respiratory failure requiring mechanical	Myocardium samples	Postmortem (4 hours after death)	Histological and immunohistochemical analysis	↑ MMP-9 compared to the control group	Smoking, hypertension, diabetes mellitus, coronary artery disease, heart failure, cancer, chronic pulmonary and kidney disease	↑ MMP-9 demonstrates the presence of myocardial inflammatory response in the myocardial tissue	https://pubmed.ncbi.nlm.nih.gov/34804033/

				ventilation							
SYED et al. 2021	Case-controlled	Race: African american, white and others; hospitalized COVID-19 (n=24), mild/moderate (n=14), and HCs (n=13)	HC: $\bar{x}$ age 56,6 years, male: n= 6, female: n= 7; mild: $\bar{x}$ 59 years, males: n= 6, females: n= 8; hosp: $\bar{x}$ 64 years, males: n= 10, females: n= 14	Mild/moderate cases, severe cases	Plasma	HCs: plasma was selected from their banked blood samples collected before COVID-19. COVID-19: Blood samples were drawn after each participant provided written informed consent from	Human active MMP-1 Fluorokine E kit	↑ MMP-1 compared to mild/moderate cases or HC	--	Both plasma levels and enzymatic activity of MMP-1 is ↑ , and its dysregulations are associated with the severity of COVID-19	https://www.ncbi.nlm.nih.gov/pmc/articles/PMC8083685/
PARRA et al. 2021	Cohort	Patients hosptalized ICU with COVID-19 (n=34), moderate COVID-19 (n=10), severe COVID-19 (n=24); Influenza H1N1 (n=23), control group (n=13 healthy donors)	Moderate COVID-19: $\bar{x}$ age 34,5 years, males: n= 7, females: n= 3; severe COVID-19: $\bar{x}$ age 19 years, males: n= 19, females: n= 5; Influenza H1N1: $\bar{x}$ age 49 years, males: n= 14, females: n= 9	Moderate and severe	Serum and plasma	At hospital admission	Luminex Assays	MMP-1 (↑ in COVID-19 compared to H1N1 and control group), MMP-2 (not mentioned) and MMP-3 (↑ in COVID-19 compared to H1N1 and control group)	Diabetes, cancer, arthralgia, myalgia, hypertension, smoking	MMP-1 and MMP-3 were associated with lung injury during COVID-19	https://www.ncbi.nlm.nih.gov/pmc/articles/PMC8115405/

ZHANG et al. 2021	Experimental	Patientes hospitalized with COVID-19 (n=53); severe (n=27), moderate (n=26), control group (n=24)	Severe COVID-19: Md 51 years, male: n= 18, female: n= 9; moderate COVID-19: Md 50 years, male: n= 17, female: n= 9; control group: Md 48 years, male: n= 14, female: n= 10	Severe and moderate	Peripheral blood mononuclear cells (PBMCs)	At hospital admission	Quantitative real-time PCR Assay (qPCR)	↑ MMP-9 compared to the control group	Diabetes, hypertension, cardiovascular disease, chronic liver disease	MMP-9 was associated with critical proteins for the function of myeloid cells.	https://www.ncbi.nlm.nih.gov/pmc/articles/PMC7930228/
EL-DIN et al. 2021	Cross-sectional	Total n= 70 participants; obese COVID-19 patients with T2DM (n=22), COVID-19 non-obese and non-diabetic patients (n= 48). Mild: n= 18 patients; moderate: n= 43; severe: n= 9.	Aged from 44 to < 55 years: n= 18; aged from 55 to < 65 years: n= 41; aged from 65 to < 75 years: n= 11; males: n= 29, females: n= 41	A mild illness, moderate and severe	Serum	COVID-19 ARDS: on the day of hospital admission. Re-avaluation: one week after hospital admission	ELISA	↑ MMP-7, ↑ MMP-9 compared to non-obese, non-diabetic group	T2D diagnosed for <10 years, obesity (body mass index ≥30kg/m²)	Lung epithelial barrier integrity is destabilized in response to the fibro-proliferative activity of elevated MMP-7 or MMP-9 and may sensitize the alveoli to a pre-fibrotic condition following SARS CoV-2 infection	https://pubmed.ncbi.nlm.nih.gov/34611417/
MOIN et al. 2021	Case-controlled interventional pilot study	Obese type two diabetes T2D (n=23) and control subjects (n=23)	Md age 62 years; group T2D: males: n= 12, females: n= 11; control group: males: n= 11, female: n= 12	--	Plasma	Measurement at baseline (control and OT2D subjects), and after 1h of insulin clamp (OT2D subjects only)	SOMA (Slow Off-rate Modified Aptamer)-scan measurements	↑ MMP-7, and MMP-9 compared to the control group	Diabetes	MMP-7 and MMP-9 ↑ indicate that lung alveolar macrophages are activated. MMP-7 has also been reported as a potential peripheral blood biomarker of idiopathic	https://www.ncbi.nlm.nih.gov/pmc/articles/PMC7979696/

										pulmonary fibrosis	
SILVA NETO et al. 2021	Prospective Study	COVID-19 GROUP (n= 237), residential group (n=60), hospitalized (n=177), and control group (n= 50 healthy volunteers)	COVID-19 Residential GROUP: x̄ age was 37; male: n= 21, female: n= 39; COVID-19 hospitalized group: x̄ age 63 years, male: n= 63, female: n= 114; control group: x̄ age 35 years, males: n= 22, females: n=28	Mild, moderate, severe, and critical	Plasma	Within 24 hours of hospital admission and 6-7 days after onset of symptom	ELISA	↑ MMP-8 in all groups compared to the control group.	Hypertension, cardiovascular disease, diabetes, smoking, stroke, neurological diseases, cancer	MMP-8 was correlated positively with sTREM-1, which means a correlation with pro-inflammatory parameters	https://pubmed.ncbi.nlm.nih.gov/34960790/
MARTINEZ-MESA et al. 2021	Cohort	Total patients with SARS-CoV-2: n= 60; 2 groups: ARDS - critically ill patients (n=29), no ARDS - those with mild to moderate pneumonia (n=31)	ARDS group, Md 67 years, males: n= 23, females: n= 6; no ARDS, Md 64 years, males: n= 5, females: n= 16	Severe: with severe pneumonia symptoms (PaO2 /FiO2 less than 200); Mild to moderate: mild symptoms of pneumonia	Plasma	At diagnosis and one week after admission	Measured by ProcartaPlex multiplex immunoassay	↔ MMP-1 and ↑ MMP-9	Arterial hypertension; diabetes mellitus; dyslipidemia; deart disease; lung disease	↔ MMP-1 levels between groups; ↑ MMP-9 levels in the ARDS group; there is a positive correlation with the risk of death	https://www.ncbi.nlm.nih.gov/pmc/articles/PMC8388961/
LERUM et al. 2021	Clinical trial	Total n= 130 participants; 3 intervention groups: treatment with	HCQ and SoC group: Md 55 years, male: n= 33, female: n= 7; SoC and Remdesivir	Admitted to the hospital ward or (ICU)	Plasma	Hospital admission and a 3-month	Duplicate by enzyme immunoassays	↑ MMP-9	Chronic lung disease, hypertension, diabetes mellitus, obesity	↑ MMP-9 during the acute phase and is associated with pulmonary fibrosis	https://www.ncbi.nlm.nih.gov/pmc/articles/PMC

		Hydroxychloroquine (HCQ) and Stardard of care (SoC) (n=40), SoC and Remdesivir (n=16), SoC (n=74)	group: Md 58 years, male: n= 15, female: n= 1; SoC: Md: 61 years, male: n=56, female: n=18			follow-up visit				compared to healthy individuals	8636497/#MOESM1
ZERIMECH et al. 2021	Longitudinal	Total n= 39 participants; 2 groups: Patients who died during (n=10), and patients alive at the end of the ICU stay (n=29)	Md living patients 60 years; males: n= 20, females: n= 9; dead patients: 58.8 years; males: n=8, females: n=8	Patients admitted to the ICU for intubation and mechanical ventilation after COVID-19	Bronchoalveolar lavage fluids (BALF) sample	Between day 1 and day 11 of ICU admission	ELISA	↔ MMP-12 compared to the patients alive at the end of the ICU	Hypertension, obesity, cardiovascular disease, dyslipidemia, apnea, chronic obstructive pulmonary disease, non-insulin-dependent diabetes mellitus	MMP-12 did not differ between deceased and living patients	https://pubmed.ncbi.nlm.nih.gov/33675194/
WU et al. 2021	Cohort	Total n= 18 participants; mild cases: n= 3, moderates: n= 3, severe: n= 1, critical: n= 8, healthy controls: n= 3	Md 57 years; moderate: males: n= 2, female: n= 1; severe: male: n= 1; critical: males: n= 7, female: n= 2; healthy controls: males: n= 2, female: n= 1	Mild, moderate, severe or critical cases	Bronchoalveolar lavage fluid (BALF) samples	From 2 to 10 days after hospitalization	Flow cytometry	↑MMP-2; ↑MMP-7; ↑MMP-9; ↑MMP-13; ↔ MMP-14 compared to mild and moderate cases	--	Severe cases: ↑ MMP-7, ↑ MMP-9, ↑ MMP-2 and ↑ MMP-13; there were no differences for MMP-14 in all groups	https://www.ncbi.nlm.nih.gov/pmc/articles/PMC8224314/
SAFONT et al. 2022	Cohort	Total n= 313 participantes; moderate (n=226), severe (n=87)	Moderate COVID 19: Md 60 years; males: n= 120, females: n= 106; severe COVID-19: Md 62 years; males: n= 64, females: n= 23	Moderate and severe	Serum	In the second and sixth months after hospitalization	The Merck Millipore kit	↑MMP-1 and ↑ MMP-7 compared to the moderate cases;	Pulmonary disease, hypertension, diabetes mellitus, cardiovascular disease, lung disease	Increased MMP-1 and MMP-7 in severe case: ; And associated pulmonary fibrosis;	https://www.ncbi.nlm.nih.gov/pmc/articles/PMC8414844/

METZEMAEKERS et al. 2021	Cohort	Total n= 121 participants, ICU patients (n= 67), and ward patients (n= 48), healthy controls (n= 6)	ICU patients were Md 62 years (50 males/17 females); ward patients was Md 69 years (26 males/22 female)	Severe cases	Plasma	Samples were collected during the first 48h after admission, one week, and at ICU discharge	ELISA	↑MMP-9 compared to the control group	Diabetes, previous myocardial infarction, congestive heart failure, arterial hypertension, peripheral vascular disease, chronic lung disease, rheumatologic disease, and kidney disease	TIMP-1/MMP-9 complexes were significantly more abundant in plasma from ICU patients; Increased levels of TIMP-1 may help to offset MMP-9 increases.	https://www.ncbi.nlm.nih.gov/pmc/articles/PMC8082714/pdf/CTI2-10-e1271.pdf
REMSIK et al. 2021	Cohort	n= 18 cancer patients hospitalized with neurologic manifestations of COVID-19	$\bar{x}$ age 64 years; males: n= 9, females: n= 9	Moderate to severe	Cerebrospinal fluid (CSF) and serum	--	ACE2 Immunohistochemical Analysis	MMP-10	Hypertension, former smoker, hyperlipidemia, diabetes mellitus, prior stroke	MMP-10 levels correlate with neuronal damage in cancer patients, positive or not for covid-19	https://www.ncbi.nlm.nih.gov/pmc/articles/PMC7833316/#mmc1
SHI et al. 2021	Cohort	Infected (n= 62), not infected (n=131), healthy (n=67), and hospitallized (n=64)	--	Infected and not infected	Serum	They were collected 3 times every 5 days after hospitalization.	Immunoturbidimetry	MMP-3	Diabetes, hematological diseases, malignant tumors, and other diseases with inflammatory reaction	There is a higher serum concentration of MMP-3 in the group infected by COVID-19	https://www.ncbi.nlm.nih.gov/pmc/articles/PMC7362036/
CHUN et al. 2021	Cohort	Total n=75 participants; home group (n= 20); non- (n=36); control group (n=7)	Md age 52 years; males: n= 34, females: n= 40	7	Plasma	On the initial study visit and 9 weeks after COVID-19 infection.	Immunoassays: MMP-9 Human-Plex and Human Cytokine	↑MMP-7, ↑MMP-8, and ↑MMP-9 compared to the control group	--	↑ MMPs in the follow-up of patients with more severe acute illness due to COVID-19, especially in patients admitted to the ICU; in particular, MMP-7 has been	https://www.ncbi.nlm.nih.gov/pmc/articles/PMC8410030/pdf/jciinsight-6-148476.pdf

										linked to strong pulmonary involvement and pulmonary fibrosis	
GELZO et al. 2022	Cohort	COVID-19 hospitalized patients (n=108), COVID-19 WHO 3 (n=52), COVID-19 WHO 4 (n=36), COVID-19 WHO 5-7 (n=20), control group (n= 48).	COVID-19 WHO 3: $\bar{x}$ age 34 years, males: n= 8, females: n= 44; COVID-19 WHO 4 $\bar{x}$ age 51 years, males: n= 19, females: n= 17; COVID-19 WHO 5-7: $\bar{x}$ age 55 years; males: n= 17, females: n= 3; control group: $\bar{x}$ age 43 years, males: n= 27, females: n= 21	WHO 3, WHO 4, and WHO 5-7	Plasma and serum	Between hospital admission and 1 week of hospitalization	Human Magnetic Luminex Assay on Biorad Bio-Plex 100 System	MMP-3: (↑ in WHO 4 compared to WHO 3 AND 5-7); MMP-9: (↔ in WHO-3 compared to the control group. ↑ in WHO 4 AND WHO 5-7 compared to control group.	Diabetes, hypertension, obesity	MMP-3 was associated with COVID-19 severity, while MMP-9 levels were associated with inflammation	https://www.ncbi.nlm.nih.gov/pmc/articles/PMC8786927/pdf/41598_2021_Article_4677.pdf
DE PAULA et al. 2021	Case-controlled	COVID-19 group: male: n= 15, female: n= 9, total n=24, compared to H1N1 group: male: n= 8, female: n=2, total n=10, and control group	COVID-19 group: Md age 71.96 years, males: n=15, females: n= 9; H1N1 group: Md age 43.5 years, male: n= 8, female: n=2; control group: Md age 42.31 years, males: n= 8, females: n=3	Hospitalization to death (control: from 8 to13 days; COVID-19: 16 days)	Lung	Postmortem (less than 2 hours)	Histological, morphometric, and immunohistochemical statistical analysis	↑ MMP-9 COVID-19 group compared to the control group. No statistical difference was observed when the COVID-19 group was compared to the H1N1 group	Bronchial asthma and interstitial pulmonary fibrosis (COVID-19 group)	↑ MMP-9 (high expression) is associated with lung injury processes. Considering the degradation of the ECM, the MMP-9 is responsible for activating TGF-β1	https://pubmed.ncbi.nlm.nih.gov/35008594/
CHAVEZ-GALAN et al. 2022	Transversal	IMV group (n=76), NIVM group (n=29), control group (n=23)	NIMV group: $\bar{x}$ age 46 years, males: n= 20 years, females: n= 9; IMV group: $\bar{x}$ age 50 years, males: n=	Severe cases	Serum	On hospital admission	ELISA Sandwich Assay	MMP-7	Obesity, smoking, diabetes mellitus, hypertension	MMP-7 level is increased only in the IMV group	https://www.ncbi.nlm.nih.gov/pmc/articles/PMC8946215/

			49, females: n= 27; control group: 5 $\bar{x}$ age 51 years								
CAMBIER et al. 2022	Cohort	COVID-19 patients (n=17), influenza patients (n=14)	COVID-19 patients: Md age 68 years, males: n= 13, females: n= 4; influenza patients: Md age 58 years, males: n= 8, females: n= 6	Severe cases	Plasma	4 to 37 days after ICU admission	ELISA Sandwich Assay	MMP-9	Diabetes, cardiovascular disease, chronic pulmonary disease, rheumatologic disease, renal disease, malignancy tumor	↔ MMP-9 activity in the different groups	https://www.ncbi.nlm.nih.gov/pmc/articles/PMC8765057/
SINGH et al. 2023	Prospective observational	mild cases: n= 23, moderates: n= 38, severe: n= 1, critical: n= 26	Md age 57 years, males: n= 61, females: n= 26;	Mild, moderate, and severe	Plasma	After ICU admission	Immunoassay assays	MMP-9	Diabetes mellitus, obesity, hypertension, renal disease, lung disease, cardiovascular disease, cancer	↑ MMP-9, -2 e -12	https://pubmed.ncbi.nlm.nih.gov/36090302/
PETRELLA et al. 2023	Transversal	COVID-19 patients: n=33, control: n=25	Md age 68,6 years, males: n= 58	Mild, moderate and severe	Serum	On hospital admission	ELISA Sandwich Assay	MMP-2 and MMP-9	Diabetes mellitus, obesity, hypertension, renal disease, lung disease, cardiovascular disease, cancer	↑ MMP-2, ↔ MMP-9	https://pubmed.ncbi.nlm.nih.gov/36831321/

ARDS: Acute respiratory distress syndrome; COVID-19: coronavirus disease 2019; ECM: Extracellular Matrix; HCQ: Hydroxychloroquine; HPT:Hypertensive; ICU: Intensive Care Unit; IMV: invasive mechanical ventilation; MMP-1: matrix metalloproteinase 1; MMP-10: matrix metalloproteinase 10; MMP-12: matrix metalloproteinase 12; MMP-13: matrix metalloproteinase 13; MvMP-14: matrix metalloproteinase 14; MMP-2: matrix metalloproteinase 2; MMP-3: matrix metalloproteinase 3; MMP-7: matrix metalloproteinase 7; MMP-8: matrix metalloproteinase 8; MMP-9: matrix metalloproteinase 9; n-HPT: non- Hypertensive; NIMV: supplemental oxygen through a nasal cannula; NS: Neurologic syndrome; sTNFR1: soluble Tumor Necrosis Factor receptor 1; sTREM-1: Soluble Triggering Receptor Expressed on Myeloid Cells-1; TDM2: Type 2 Mellitus Diabetes; TGF- b1: Transforming Growth Factor-beta1; TIMP-1: Tissue Inhibitor of Metalloprotease-1; TNF-α: Tumour Necrosis Factor alpha; WHO: World Health Organisation; $\bar{x}$= mean; Md: median.
